# A new conservation material for gold in heritage wall paintings: polymer-stabilized nanogold gels (NGGs)†

**DOI:** 10.1039/d4na00877d

**Published:** 2024-12-11

**Authors:** Maram Na'es, Lars Lühl, Birgit Kanngießer

**Affiliations:** a Institute for Optics and Atomic Physics, Technical University Berlin Hardenbergstr. 36 10623 Berlin Germany maram@physik.tu-berlin.de

## Abstract

Gilded wall paintings such as those in Petra-Jordan undergo deterioration processes such as delamination and loss of the gold layer. The aim of this work is to produce a functioning long-lasting adhesive that compensates for binder and gold loss while stabilising the gold layer. Polymer-stabilised gold nanoparticles (AuNPs) as a conservation material for gilded heritage paintings (Nano Gold Gel (NGG)) were synthesised using two facile and affordable synthesis approaches. AuNPs enhance the stability of the adhesive polymer over time and introduce mass conservation to the gold layer. Two natural polymers and one synthetic polymer, frequently used in conservation as adhesives, were used as reducing agents and stabilisers for the nanoparticles. The chemical alteration of the polymers and the Au–polymer interaction at the molecular level were investigated with FTIR spectroscopy, while the chemical environment of gold was investigated with X-ray absorption spectroscopy (XANES/EXAFS). The synthesized NGG was applied on the replica samples to reattach the gold layer to its support. Characterisation results indicate that the formation of AuNPs stabilised by the three polymers did not alter the chemical structure of the polymers. The applied NGG successfully achieved re-adhesion and exhibited appropriate optical and chemical properties for use as a conservation material.

## Abstract 2

Gilded wall paintings continue to lose their gold layer over time. A new polymer-stabilised gold nanoparticles adhesive (NGG) was produced and tested. It proved successful re-adhesion and appropriateness as a conservation material.

## Graphical abstract

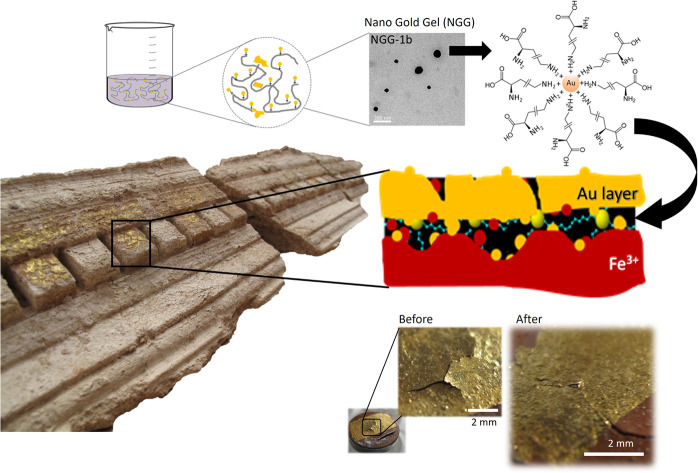

## Introduction

Gilded wall paintings and stucco often undergo various deterioration processes.^1–3^ Gold delamination is particularly a common phenomenon due to binder aging and loss.^1^ One typical conservation treatment is the overlaying of the original gold layer with a new gold leaf or powder fixed using an organic binder. The elemental composition of commonly used commercial gold conservation materials shows a high copper content which is not recommended due to its reactivity with chloride and sulfate ions in the surrounding environment.^4,5^ This leads to accelerated chemical degradation of gold which is unwanted in a conservation treatment. Therefore, new conservation protocols and materials need to be considered. In the case of gold delamination, compensation for lost gold and binder is needed to be comparable quantitatively and compositionally with that in the historical samples. A small-size form of gold mixed with a long lifetime binder to be delivered underneath the delaminated gold layer is a viable solution. The use of gold nanoparticles (AuNPs) enhances antibacterial activity which wall paintings sometimes suffer from^6^ and provides stability and long-lifetime for the final adhesive.^7^ Therefore, multiple Nano Gold Gels (NGGs) composed of AuNPs stabilized with variable polymers compatible with gold conservation, were synthesized.

The synthesis of AuNPs as comprehensively reviewed by Zhao (2013),^8^ shows that *in situ* preparation by chemical reduction involves two major steps: reduction and stabilization. The chemical nature of the main amino acid part of the natural polymers of proteins and biomolecules, such as gelatin and gum arabic, proved adequate in functionalizing as both stabilizing and reducing agents when added to gold precursors.^6,8–14^ A wide range of applications from medicine, food, and cosmetics are well reported.^8^ However, the application of nanoparticles in the field of cultural heritage is so far known for deacidification, consolidation, and cleaning.^15–17^ Those were reviewed in 2006 (ref. 16) and reattachment of paint layers has not yet been an application.

In this work, we present the use of polymer-stabilized gold nanoparticles for reattaching the gold layer to its support. Two natural polymers were used as reducing and stabilizing agents in the synthesis of gold nanoparticles. Au_2_O_3_ and HAuCl_4_ were used as gold precursors. Production of this metal–polymer gel was additionally experimented on the commonly used adhesive in conservation Paraloid B72, which is a synthetic acrylic copolymer of ethyl methacrylate (70%) and methyl acrylate (30%).^18^ The synthesized NGGs were then applied on gilded replica samples to test their performance as a conservation material.

## Materials and methods

### Materials

Deionized water with 18 MΩ cm resistivity was used for all experiments. Gold(iii) chloride trihydrate (HAuCl_4_·3H_2_O), trisodium citrate (Na_3_C_6_H_5_O_7_·2H_2_O), gold(iii) oxide dihydrate (Au_2_O_3_·2H_2_O), and gold(i) sulfide (Au_2_S) were purchased from Sigma-Aldrich and were of analytical grades (>99.9%). Gelatin sheets (ID 63053), gum arabic powder (ID 63330), and Paraloid B72 (ID 67400) were purchased from Kremer Pigmente®, Germany. Acetone (Sigma-Aldrich, Analytical Grade) was used to dissolve Paraloid B72. Stock solutions of 3.3% wt/wt gelatin, 3% wt/wt gum arabic, 3% wt/vol ethyl methacrylate copolymer (Paraloid B72), 10 mM HAuCl_4_·3H_2_O, and 2.2 mM Na_3_C_6_H_5_O_7_·2H_2_O were prepared; more details are provided in ESI Table S1.†

### Synthesis approaches

Two synthesis approaches were used to produce NGGs. The first is a green approach using mechanochemistry and one-step biochemical synthesis. The second is a chemical approach based on the Turkevich method followed by citrate substitution.

### Green synthesis of nano gold gels (NGGs)

Self-prepared shell gold from Doppel gold leaf was prepared with gelatin in one approach and with gum arabic in another. The first approach is mechanochemical using a pestle and mortar, and the second is a one-step biochemical synthesis *via* Au_2_O_3_ and HAuCl_4_ precursors. The main synthesis parameters including gold precursors, reducing and stabilizing agents, and reaction conditions are provided in Table 1.

**Table 1:** Synthesis parameters for the green biochemical approach

NGG	Gold precursor	Reducing and stabilizing agent	Reaction conditions
NGG-1b	HAuCl_4_·3H_2_O (10 mM, 5 mL)	Gelatin (3.3%, 5 mL)	80 °C, vigorous stirring, 90 minutes
NGG-2b	HAuCl_4_·3H_2_O (10 mM, 5 mL)	Gum arabic (3%, 5 mL)	80 °C, vigorous stirring, 90 minutes
NGG-3b	HAuCl_4_·3H_2_O (10 mM, 5 mL)	Paraloid B72 (3%, 5 mL)	RT, vigorous stirring, 90 minutes
NGG-1c	Au_2_O_3_ (1 mg)	Gelatin (3.3%, 1 mL)	80 °C, vigorous stirring, 90 minutes
NGG-2c	Au_2_O_3_ (1 mg)	Gum arabic (3%, 1 mL)	80 °C, vigorous stirring, 90 minutes
NGG-3c	Au_2_O_3_ (1 mg)	Paraloid B72 (3%, 1 mL)	RT, vigorous stirring, 90 minutes

### Chemical synthesis of nano gold gels (NGGs)

AuNPs were synthesized using the Turkevich method^8^ (eqn (1)) where chloroauric acid as the gold precursor and sodium citrate as the reducing and stabilizing agent are used. Re-stabilization of the synthesized Au-NPs *via* citrate-substitution with gelatin, gum arabic, and Paraloid B72 was then employed. 5 mL of 2.2 mM sodium citrate solution (Na_3_C_6_H_5_O_7_) were added to 5 mL of boiling solution of 10 mM HAuCl_4_·3H_2_O under rigorous stirring for 25 minutes. The color change of the reaction solution was observed from colorless to pink to light red until it reached a ruby red color. Proteins and other biomolecules can associate with nanoparticles and stabilize them through binding to their surfaces. The citrate molecules are weakly adsorbed on the surface of AuNPs^9–14^ which allows ligands with higher binding affinity to substitute them, hence making nanoparticle functionalization relatively easy. The principle of the synthesis follows the work of Neupane (2011).^11^ The produced AuNPs were used as synthesized. The main synthesis parameters including gold precursors, reducing and stabilizing agents, and reaction conditions are provided in Table 2, while detailed synthesis information can be found in ESI Section S2.†

1 2AuCl_4_^−^ + Ctr_3_^−^ + 2H_2_O → 2Au + 3CH_2_O + 3CO_2_ + 8Cl^−^ + 3H^+^

**Table 2:** Synthesis parameters for the chemical approach

NGG	Gold precursor	Reducing and stabilizing agent	Reaction conditions
NGG-1d	Citrate stabilized Au-NPs (5 mL)	Gelatin (3.3%, 5 mL)	80 °C, vigorous stirring, 90 minutes
NGG-2d	Citrate stabilized Au-NPs (5 mL)	Gum arabic (3%, 5 mL)	80 °C, vigorous stirring, 90 minutes
NGG-3d	Citrate stabilized Au-NPs (5 mL)	Paraloid B72 (3%, 5 mL)	RT, vigorous stirring, 90 minutes

### Preparation of *in vitro* samples

Both NGG-1b and NGG-1c (100 μL each) were deposited on a Doppel gold leaf (Kremer Pigmente GmbH & Co., ID 98412), α-Fe_2_O_3_ (Kremer Pigmente GmbH & Co., ID 48120), Fe-rich clay (SRM 679, NIST), and gypsum (Sigma-Aldrich, ID 12090) (ESI, Table S1†) to resemble paint, mordant, and preparation layers, respectively, of the gilded structure. An illustrative description of *in vitro* applications can be found in ESI Scheme S1.†

### Characterization and assessment methods

**Fig. 1:**
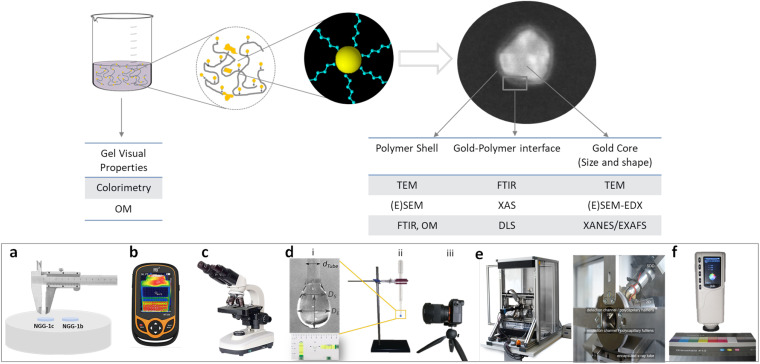
(Top) Illustration of the characterization approach and methods for the synthesized NGGs. (Bottom) Assessment methods for NGG application *in vitro*. (a) Direct surface-spread measure, (b) thermal imaging, (c) optical microscopy, (d) surface tension measurements (i) magnification of the drop and the parameters used in evaluating surface tension, (ii) Pasteur pipette, (iii) Canon G10 camera fixed on a tripod to guarantee image capturing stability and repeatability, (e) 3D-μXRF back and front views, and (f) colorimetry analyzer and the color reference card.

Characterization of optical, physical, and chemical properties of the synthesized NGGs was performed using the approach and methods illustrated in Fig. 1. Particle size and shape of the produced AuNPs were determined using TEM and ESEM, and the hydrodynamic diameter (*d*_h_) using DLS. Optical microscopy (OM) was also used to visualize the gel system. The chemical interaction at the gold–polymer interface was investigated by FTIR analysis and chemical speciation of Au using XANES/EXAFS at the Au-L_3_ edge. The latter was also used to assess the production yield of selected synthesized NGGs adhesives. Penetration depth, lateral distribution, and surface tension of the two selected NGG adhesives applied on replica samples of the four interfaces of the gilded structure were used to assess them as conservation adhesives *in vitro*. A brief description of the used methods is given below while technical details and settings can be found in ESI Section S3.†

Au-L_3_ XANES and EXAFS measurements were performed at the mySpot beamline of the BESSY II synchrotron.^19^ The beamline is located at a 7 T wavelength shifter providing X-ray energies up to 30 keV. The beam is pre-focused by a mirror situated close to the X-ray source and is monochromatized using two pairs of crystal monochromators: Si (111), *λ*/Δ*λ* ∼ 5000 and a double-crystal mirror with MoB_4_C coating, *λ*/Δ*λ* ∼ 30 allowing energy resolution of Δ*E*/*E* = 1.25 × 10^−4^. A pinhole of 100 × 100 μm^2^ is placed in front of all samples. Measurements were performed in fluorescence mode where the fluorescence radiation of the sample is detected in reflection mode using a 7-element SiLi detector positioned at an angle of 90° with respect to the excitation beam. Au-L_3_ XANES spectra of gold reference materials were collected in transmission and fluorescence modes (see ESI, Table S3†). For FTIR analysis, an Agilent 4300 handheld spectrometer was used (spectral range 4000–650 cm^−1^, DTGS detector, 600 scans, 4 cm^−1^ spectral resolution, 4 zero filling factor, Happ-Genzel apodization). Samples with rough surfaces and low reflection were analysed using the DR interface (DRIFT), while miniature samples were analyzed with the ATR interface. DLS measurements were carried out on a static/dynamic compact goniometer (SLS/DLS-5000), ALV, Langen Germany. A He–Ne laser with 22 mW power was used for dynamic light scattering with scattering angles between 30° and 100° at 5° intervals and 45 s measuring time at each angle. The sample temperature was regulated to 293.1 ± 0.1 K. For SEM-EDX analysis, samples were carbon-coated and analysed using a JEOL JSM-840 (Tokyo, Japan) with a LINK AN 10000 microanalyser. Micrographs (4096 × 4096 pixel size) were acquired in backscattered mode, EDX analysis was performed with 20 keV acceleration voltage and a beam current of 1–3 × 10^−4^ mA for 100 s per analyzed point. Microimaging under low controlled pressure was performed directly on the samples using an Uwe Binninger Analytik ESEM without any prior preparation. TEM measurements were performed using a Jeol JEM 2200-FS operating at 200 keV. Confocal 3D-μXRF analysis was carried out using a 30 W X-ray tube (Mo target), an energy-dispersive SSD detector, and two X-ray polycapillary lenses located at 90° geometry with both excitation and detection channels being at 45° with the sample surface normal. A full polycapillary lens is mounted in the excitation channel (22 μm focal spot size at 7.5–10 keV), and a half lens in the detection channel. A palm-size portable 3nh Precision Colorimeter, Model NR10QC, was used for colorimetry measurements acquired in reference to the CIE L*a*b*^20^ chromaticity diagram and using the color chart standards of B.I.G. The setup allows an 8° illumination angle and a diffuse reflection component through a measuring aperture of *φ* 4 mm and *φ* 8 mm. The measured area is illuminated with the standard illuminant D65 LED blue light and reflected light is detected with a photoelectric diode. Color differences Δ*E* were calculated from the collected color coordinates for each measured point using the CIE 1976 equation (see the ESI†).

## Results and discussion

### Characterization of NGGs

#### Gold nanoparticles: shape, size, and optical properties

**Fig. 2:**
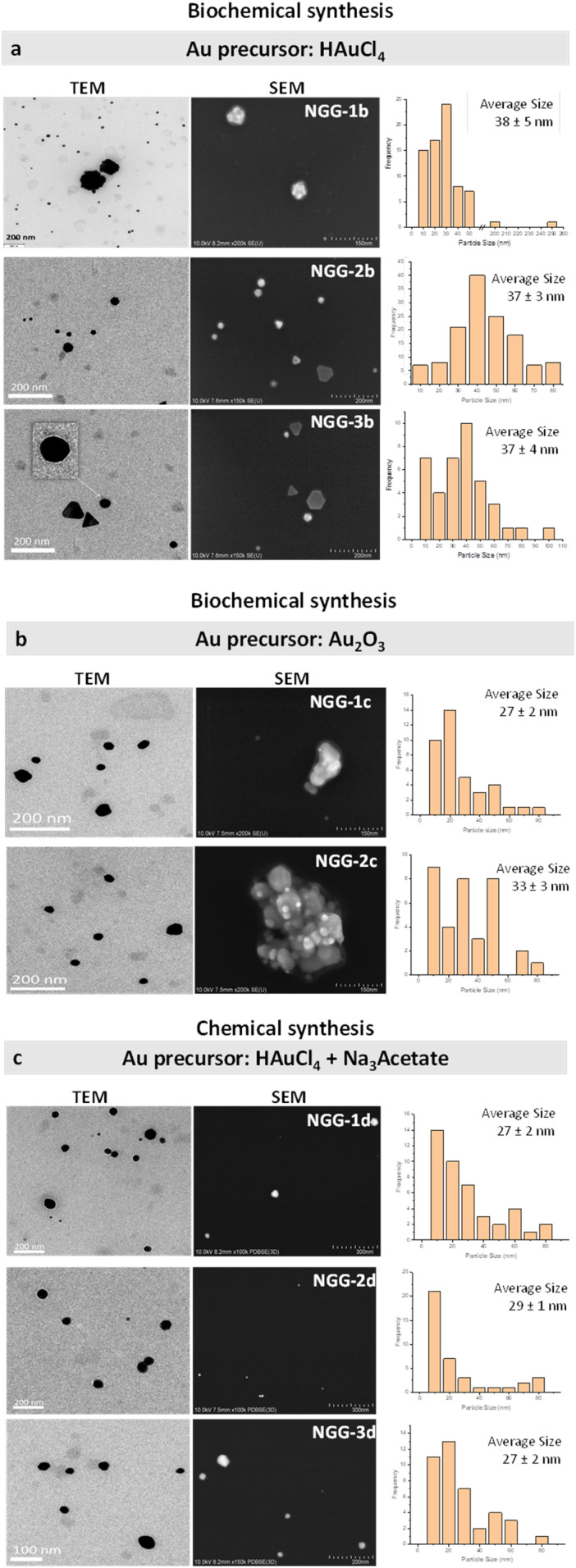
SEM and TEM characterization and particle size histograms of a selection of the synthesized NGGs categorized according to the synthesis method and the Au precursor (a) synthesized NGGs from the HAuCl_4_ precursor (from top to bottom): NGG-1b, NGG-2b, and NGG-3b. (b) Synthesized NGGs from the Au_2_O_3_ precursor (from top to bottom): NGG-1c and NGG-2c. (c) Synthesized NGGs from HAuCl_4_ and trisodium acetate using the Turkevich method (from top to bottom): NGG-1d, NGG-2d, and NGG-3d.

The results of the microscopic characterisation of the synthesised NGGs are shown in Fig. 2. The mechanochemically synthesized NGGs (NGG-1a and NGG-2a) adhesives show large-size gold nanoparticles with irregular shapes and forms which more correctly can be described as gold nanoflakes. Biochemically synthesized NGGs *via* chloroauric acid (NGG-1b, NGG-2b and NGG-3b) have similar particle size with an average of 37.3 nm ± 4 nm, although particle size distribution varies for the three NGGs. NGG-1b shows higher frequency at 30 nm size and below, while NGG-2b shows a more Gaussian distribution. NGG-3b shows competitive frequency at small and large particle sizes with a maximum occurrence at 40 nm. Particles as large as 100 nm were also found in this case. As revealed by ESEM, SEM, and TEM analyses, the used polymer did not influence the formation of AuNPs, and successful synthesis was achieved using the three polymers. However, particle shape varied slightly between the three NGGs. Round to spherical multi-core particles were formed using gelatin polymer (NGG-1b). Aggregation was also visible in this sample as highlighted in the inset of the TEM micrograph. Closer inspection of NGG-2b and NGG-3b indicates the formation of a mixture of more regular shapes including triangular, hexagonal, and spherical, in addition to multi-core particles. Both NGGs have an average particle size of 37 nm ± 3 nm. Polymer capping of AuNPs was also visible at high magnification and resolution of TEM (Fig. 2).

As repeatedly reported in the literature,^8,10,12,13,21–25^ reaction conditions influence the shape and size of synthesised AuNPs. Reaction time and temperature are most importantly considered in the case of organic polymers as reducing and stabilising agents. It is possible to improve homogeneity of particle size and shape of the synthesised NGGs. However, considering the purpose for which these materials are meant to be used for, it is not critical to have heterogeneous size and shape of AuNPs. It is more essential to have a functioning long-lasting adhesive which compensates for binder and gold loss and stabilises the gold layer.

The biochemical synthesis *via* gold(iii) oxide, gelatin and gum arabic succeeded in reducing and stabilising gold leading to the formation of NGG-1c and NGG-2c, respectively, while there was no formation of AuNPs when Paraloid B72 was used. ESEM and SEM analyses show that both natural polymers of gelatin and gum arabic form continuous gel layers trapping AuNPs within. The particle shape of AuNPs in both NGGs does not appear different from one another, nor highly geometrical. It rather appears amorphous with intense aggregations up to the micro-scale, particularly in the case of gum arabic. Diluting the concentration of both NGGs using more amount of their respective polymer, encourages particles to disperse and dis-aggregate. This is seen in the small particle size with an average of 27 nm ± 2 nm for NGG-1c, and 33 nm ± 3 nm for NGG-2c (Fig. 2).

Chemically synthesized NGGs *via* the Turkevich method using chloroauric acid and trisodium citrate salt then replaced and restabilized with the three polymers (NGG-1d, NGG-2d and NGG-3d) have comparable particle size with an average of 27.7 nm ± 2 nm, although particle size distribution profiles vary for the three NGGs. NGG-1d shows a maximum frequency at 10 nm size that decreases gradually as particle size increases. NGG-2d shows a majority of 10 nm size particles. In contrast, NGG-3d has a wider size distribution profile with more frequency of smaller sizes at 20 nm. The synthesized AuNPs have mostly spherical shapes regardless of the used polymer. No aggregation is noticed in this synthesis approach. Optical microscopy shows a light purple color for all NGGs. The characteristic cell-like polymer structure of Paraloid B72 can still be seen in NGG-3d suggesting the intact polymer structure. Synthesized NGGs are meant to be applied between gold and iron-oxide layers underneath. No visual residue is meant to be left, at least not visually. The color of the iron oxide layer in historical samples was mostly orange or red, yellow was found in a few instances. The visible color of produced NGGs varied between very light red, red, and light purple. It is to be mentioned that redispersion of AuNPs in the produced NGGs using the same polymer used in the relevant synthesis approach, remains possible when viscosity and color need to be adjusted. This step will reduce AuNP concentration, and this needs to be considered in advance.

#### Gold chemical speciation in produced NGGs

Since the energy position of Au-L_III_ absorption edge is highly dependent on gold's oxidation state, XANES and EXAFS analyses were performed on the Bessy II synchrotron at Helmholtz Zentrum Berlin (HZB), to identify gold oxidation in the synthesized gold nanogels. Absorption spectra were background subtracted followed by standard normalization procedures for both excitation energy intensity and lifetime. Selected gold reference materials were also investigated for calibration purposes and for use in Linear Combination Fit (LCF) in the ATHENA program.^26^

**Fig. 3:**
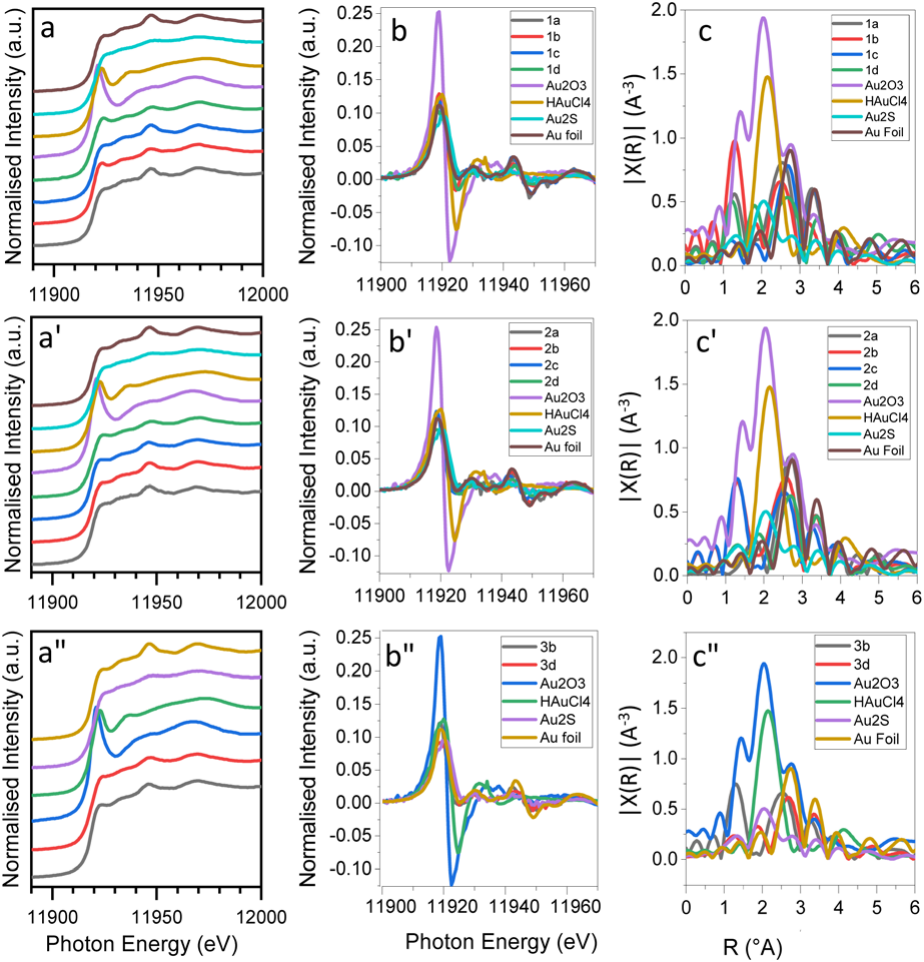
Au-L_3_ XANES spectra, their 1^st^ derivative, and the magnitude of the Fourier transforms of the *k*^2^-weighted EXAFS spectra for (a–c) gelatin stabilized NGGs, (a′–c′) gum arabic stabilized NGGs, and (a′′–c′′) Paraloid B72 stabilized NGGs, plotted against reference materials of metallic gold, gold(iii) oxide, and HAuCl_4_, and Au(i) sulfide.

Au-L_3_ XANES spectra of the synthesized NGGs are presented in Fig. 3 according to their stabilizing polymer. Both NGG-1a and NGG-1c show main spectral features characteristic of Au(0), while NGG-1b and NGG-1d show more HAuCl_4_ features in the edge region (Fig. 3a–c). However, the rest of the spectral features do not match those of HAuCl_4_. Instead, they return to match Au(0). Linear Combination Fit (LCF) results (see the ESI†) show the contribution of both Au_2_O_3_ and HAuCl_4_ in the synthesized NGG. It is to be noted that both NGG-1b and NGG-1d were produced using HAuCl_4_ as the gold precursor, which was also detected by FTIR characterization in the next section. The magnitude of the Fourier transforms of *k*^2^-weighted EXAFS spectra (Fig. 3c) shows mostly Au(0) features for NGG-1a, NGG-1c, and NGG-1d, while NGG-1b shows combined features of Au(0) and Au(iii), particularly Au_2_O_3_ and HAuCl_4_. Apparently, XANES white line features are influenced by the metal nature being bulk or nano-size. These results are in good agreement with earlier studies investigating nanogold compounds.^27,28^

XANES analysis of gum arabic stabilized NGGs are shown in Fig. 3a′–c′. The synthesized NGGs (NGG-2a, NGG-2b, NGG-2c, and NGG2d) show similar spectral features to Au(0). A few, weak, and irregular variations in intensity in the edge region are noticed. These resemble the influence obtained when analyzing nanoparticles.^27,28^ Examining the first derivative for this NGG batch shows that all four NGGs (NGG-2a, NGG-2b, NGG-2c, and NGG2d) produced using gum arabic have zero oxidation state for gold. However, variable percentages of the Au(0) component are obtained from LCF of the XANES spectra. The presence of gold chloride and oxide features arise from impurities in the final product as also detected by FTIR (see the next section). As shown in Fig. 3c′, NGG-2b and NGG-2c appear to have combined features as seen earlier for NGG-1b.

XANES spectra of Paraloid B72-stabilised nanogels (NGG-3b and NGG-3d) and their first derivatives (Fig. 3a′′–c′′) show mainly spectral features of Au(0). Meanwhile the magnitude of the Fourier transforms of *k*^2^-weighted EXAFS spectra for both NGGs shows that NGG-3b has similar combined features, unlike NGG-3d which appears in good agreement with Au(0) metal as shown in Fig. 3c′′. In contrast, LCF shows components of Au(iii) compounds. Absorption edge energies of the synthesized NGGs (see the ESI†) indicate good alignment with the Au(0) edge energy.

#### Gold–polymer bonding in produced NGGs

**Fig. 4:**
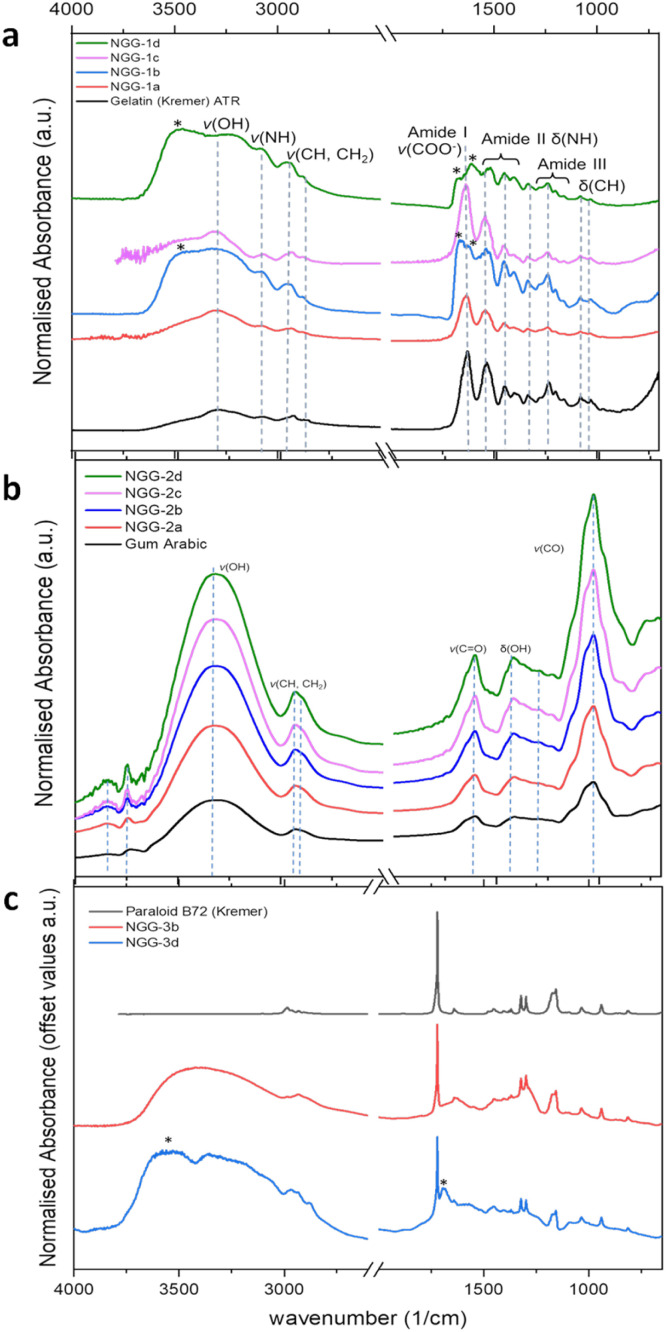
FTIR spectra of (a) gelatin stabilized NGGs (NGG-1a, NGG-1b, NGG-1c and NGG-1d), (b) gum arabic stabilized NGGs (NGG-2a, NGG-2b, NGG-2c, and NGG-2d), and (c) Paraloid B72 stabilized NGGs (NGG-3b and NGG-3d) together with their corresponding stabilizing polymers.

Infrared analysis was used to investigate chemical bonding between gold and polymers by tracing vibrational change of main functional groups of the stabilizing agents in the produced NGGs. Dry films of produced NGGs and reference materials were investigated using ATR-FTIR. Spectral features of pure gelatin and all four NGGs (Fig. 4a) remain intact, indicating no chemical change in gelatin polymer chains has occurred upon stabilization of the gold nanoparticles. A few changes in some features are however observed. A change in band intensity and a slight energy shift at 3080 cm^−1^ (N–H stretching) are observed for NGG-1b and NGG-1d indicating possible coordination between amine groups and AuNPs. For both NGGs, the C=O stretching of amide I at 1650 cm^−1^ appears to have a slight shift and combination with another new band at around 1680 cm^−1^. A citrate band appears in this region indicating that citrate capped AuNPs are still present in the final nanogel, *i.e.* imperfect purification.

FTIR results show that the use of gelatin as a stabilising agent for AuNPs does not alter its polypeptide backbone and side chain functional groups through mechanochemical synthesis and biochemical synthesis processes *via* the Au(iii) oxide precursor. For the other two synthesis processes, a few alterations are noted which suggest possible chemical interaction with AuNPs. This supports the idea of gelatin functionality as a stabilising agent for AuNPs through its steric protection and bulky protein feature. The results are also in agreement with Neupane^11^ for characterising gelatin–AuNPs bonding.

FTIR analysis results of gum arabic stabilized NGGs (NGG-2a, NGG-2b, NGG-2c, and NGG-2d) are presented in comparison with a reference material of gum arabic (Fig. 4b). For the synthesised NGGs (NGG-2a, NGG-2b, NGG-2c, and NGG-2d), results are in agreement with Dhar (2008)^10^ where no spectral changes are noticed indicating no alteration of the polymer. However, this does not necessarily mean there is no chemical interaction between gum arabic and AuNPs. Rather, gum arabic shows no chemical alteration in its structure when used as a stabilising agent for AuNPs synthesised through the four approaches. FTIR analysis of Paraloid B72 stabilized NGGs (NGG-3b and NGG-3d) are presented in Fig. 4c together with a reference sample of Paraloid B72. The spectra show the presence of citrates in the final product, as marked with asterisks in Fig. 4c, indicating that excess of citrate-stabilized AuNPs is co-present with NGG-3d. According to state-of-the-art research, there is no study reporting the use of Paraloid B72 to stabilise AuNPs, nor any other synthetic adhesive polymer used for conservation purposes. The results show predominant spectral features of Paraloid B72 functional groups, indicating the preservation of its structure over both synthesis regimes. Both characteristic C=O stretching bands at 1720 cm^−1^ and 1640 cm^−1^ remain intact and unshifted in both spectra of NGG-3b and NGG-3d. However, two bands at 1694 cm^−1^ and 3600 cm^−1^ appear for NGG-3d. As previously seen with chemically synthesized NGGs, a contribution from citrate capped AuNPs is evident. This supports the conclusion that the purification process of the final NGG requires improvement, and partial citrate substitution seems to have taken place.

### Assessment of NGGs as conservation adhesives

#### Chemical interaction of NGGs with gilding individual interfaces

**Fig. 5:**
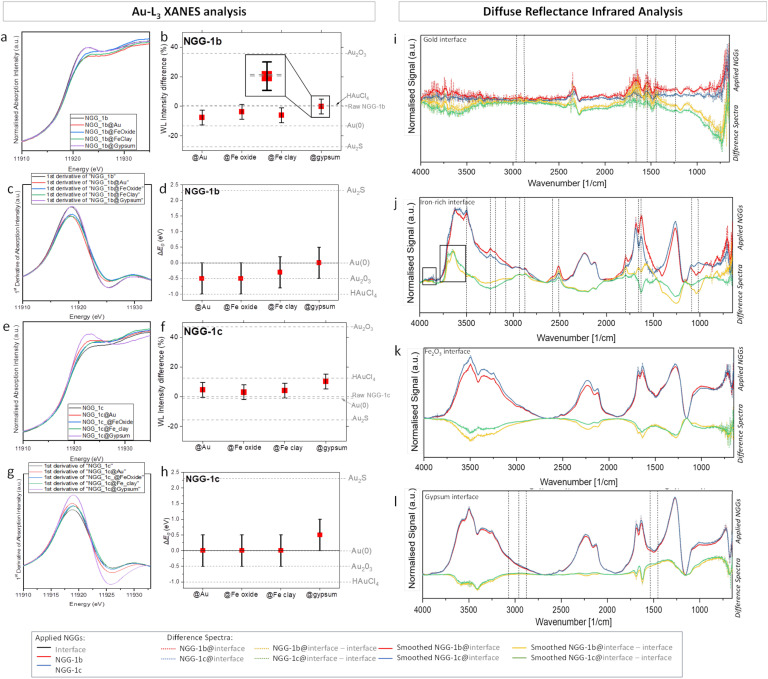
Au-L_3_ XANES spectra of pure and applied (a) NGG-1b and (e) NGG-1c, at the four investigated interfaces. Difference in white line intensity of applied (b) NGG-1b and (f) NGG-1c in reference to their raw gels. WL intensities of gold standards are marked in grey. XANES first derivative of pure and applied (c) NGG-1b and (g) NGG-1c. Shift of absorption edge energies of applied (d) NGG-1b and (h) NGG-1c at the four interfaces in reference to their raw gels. *E*_0_ of gold standards are marked in grey for comparison. (i–l) DRIFT spectra of applied gels and their difference spectra at the gold, iron-rich clay, Fe_2_O_3_, and gypsum interfaces, respectively.

To understand the interaction between the synthesized conservation materials and the gilded surface, assessment studies were limited to two nanogold gels NGG-1b and NGG-1c with the same adhesive polymer and different gold precursors, as representatives for the behavior of the new adhesives. This has additionally allowed us to study the impact of gold precursors on the behavior of the synthesized adhesive as a conservation material. Each adhesive was applied on the four individual interfaces composing the gilded structure, namely: gold, iron(iii) oxide, iron-rich clay, and gypsum. This application is called here ‘*in vitro*’. The interaction was assessed using XANES analysis at the Au-L_3_ edge in fluorescence mode (Fig. 5a–h) and infrared analysis in reflectance mode (DRIFT) (Fig. 5i–l). Beamtime allocation for XANES and EXAFS measurements allowed the assessment study of only two NGGs.

LCF of XANES spectra and a quantitative comparison of absorption edge energies *E*_0_ and white line (WL) intensities for pure and applied gels were used to describe spectral differences. It is to be noted that all the values of *E*_0_ shifts in the studied samples are equal to or less than 0.5 eV, which is the best experimentally achievable energy resolution using the Si (111) monochromator at the beamline.^29^ XANES spectra and their corresponding WL intensity values, first derivative spectra, and energy shift values are presented for NGG-1b and NGG-1c as dry films of pure gels and as applied gels over the four interfaces. Values for gold standards are shown in horizontal grey lines to highlight spectral differences in white line intensity and energy shift of the absorption edge. As can be seen, oxidation of gold increases WL intensity and decreases absorption edge energy (Fig. 5b, d, f and h).

As shown in Fig. 5a–d, when NGG-1b (*E*_0_ 11 919.0 ± 0.5 eV) is applied on the bulk gold interface and α-Fe_2_O_3_ interface, an energy shift of 0.5 eV each towards lower absorption energy is seen. Similarly, the WL intensity of both spectra decreases compared to raw NGG-1b (7.7% and 3.8%, respectively). The interface spectrum of NGG-1b with iron-rich clay shows about 6% decrease in WL intensity and a slightly lower *E*_0_ shift compared to the first two interfaces (−0.3 eV). The gypsum interface with NGG-1b, however, shows no energy shift in the absorption edge and a negligible difference in WL intensity (0.06%). At the first three interfaces, a reduction in WL intensity is observed while absorption edge energy shifts towards lower values compared to raw NGG-1b. The first observation indicates more spectral features of bulk gold (Fig. 5b), while the latter indicates more spectral features of an oxidised state of Au, specifically Au(iii) oxide (Fig. 5d). This contradiction can be attributed to electron density screening of gold nanoparticles upon their interaction with the interface material. According to previous studies,^30,31^ the electron density screening is influenced by the electron density of the stabilising agent (gelatin in this case) and by the electronegativity of support's main metal centres, which are Au, Fe, Ca, and S in the case of the investigated interfaces. Such screening often influences the XANES region and lowers the absorption edge energy.^31–36^ This effect appears clearer in metal nanoclusters and particles compared to their bulk forms.^30,31,36,37^ Charge transfer phenomena between nanoparticles and their support are also known to affect the XANES region.^30^ Since the nanogold material is not the changing parameter then it must be its interaction with the support that influences the surrounding environment of gold as probed by XANES. Formation and agglomeration of Au-nanoparticles upon irradiation with X-rays is also known to occur under high radiation flux such as that of a synchrotron source.^30^ This also influences the spectra to exhibit more bulk gold spectral features in terms of lower WL intensity. This is actually evident in the preparation of metallic gold nanoparticles from aqueous solutions in the presence of a stabilizer without using chemical reducing agents,^38^ for example by using the radiolytic method.^39–42^ In contrast, when NGG-1c (*E*_0_ 11 919.0 ± 0.5 eV) is applied over the four interfaces no energy change is observed for the absorption edge of the first three interfaces of bulk gold, α-Fe_2_O_3_, and iron-rich clay (Fig. 5e–h). However, their corresponding white line intensities (+4.7, +2.9, and +4.1%, respectively) show a slight increase compared to that of raw NGG-1c (Fig. 5e and f). Moreover, *E*_0_ for NGG-1c over gypsum shows a blue shift of 0.5 eV and an increase in WL intensity (10.2%), indicating a possible formation of partially charged gold species (Au^+*δ*^).

#### Molecular assessment at individual interfaces

Changes at the molecular level of NGG-1b and NGG-1c conservation materials before and after application over the four inspected interfaces were assessed using DRIFT analysis. Difference spectra between applied NGGs at the four interfaces and interface's raw spectra (applied over the gypsum support) were used to extract spectral features and changes due to NGG application. These are shown in Fig. 5i–l at the interfaces of gold, iron oxide, iron-rich clay, and gypsum, respectively.

In the difference spectra discussed below, the negative difference means support features are prevailing over the NGG, while positive difference means NGG is molecularly detectable. The latter can indicate minimal penetration of NGG in the studied interface layer and accumulation at the surface. Inverted bands and first derivative bands (due to Reststrahlen) appear positive and are identical to raw support spectra.

When NGG-1b and NGG-1c spectra at the same interface do not match, it indicates a different interaction with the interface material. This confirms that gelatin binds differently to Au depending on the preparation method, as seen at Fe_2_O_3_ and gypsum interfaces.

It is important to mention two main analytical differences between assessment methods with X-ray and infrared radiation. First is the spot size which is 6 ± 1 mm for DRIFT and 12 μm (10–15 keV, 45° geometry) for XANES measurements. Second is the penetration depth which is 1–3 μm for DRIFT and 5–20 μm for XANES analysis. This means, DRIFT analysis is a more surface sensitive technique compared to XANES analysis. Hence, more surface information is obtained using DRIFT analysis over a much wider surface area compared to that of XANES. However a narrower area with deeper penetration depth into the sample subsurface layers is obtained using XANES analysis.

#### Molecular assessment at the interface with Au

At the NGG-gold interface, the gold layer is expected to reflect all incident infrared radiation. However, atmospheric and surface material interfere with final spectra as observed in Fig. 5i. Both NGG-1b and NGG-1c behave similarly and show no detectable spectral change after interaction with the interface, as also suggested by LCF results (ESI, Fig. S5†). A few peaks are observed at 1238, 1443, 1537, 1630, and 1660–1668 cm^−1^ mainly originating from protein interferences which match protein peaks of the NGG stabiliser. However, no aliphatic chain bands around 3000 cm^−1^ or higher are visible indicating a likely penetration of NGGs through the gold leaf and down to the gypsum support. Characteristic bands of the gold–gypsum support are prevailing as seen in the negative spectral difference.

#### Molecular assessment at the interface with iron α-Fe_2_O_3_

Both NGG-1b and NGG-1c behave similarly and show no detectable spectral change upon the interaction with α-Fe_2_O_3_ (Fig. 5j). Characteristic bands of the iron oxide–gypsum support prevail strongly and hide NGGs peaks, this hinders detecting any molecular change occurred to NGGs over iron oxide, if any. Difference spectra show mainly the inverse of thr α-Fe_2_O_3_–gypsum support, indicating possible penetration of NGGs through the α-Fe_2_O_3_ layer down to the gypsum layer. LCF of XANES spectra (ESI, Fig. S6†) shows good agreement with DRIFT results where no detectable alteration to both NGGs is observed.

#### Molecular assessment at the interface with Fe-rich clay (SRM 679, NIST)

Both NGG-1b and NGG-1c behave almost similarly after interaction with the iron-rich clay interface and show intensity variation in main clay–gypsum support peaks (Fig. 5k). At 1019 cm^−1^, raw and applied NGG-1b have lower intensity than raw and applied NGG-1c. Unlike the peak at 1088 cm^−1^ where applied NGG-1b increases intensity to match that of applied NGG-1c. Gypsum sulfate appears at 1100 cm^−1^, and the change that occurred for NGG-1b at 1088 cm^−1^ is likely indicative of chemical bonding with the gypsum sulfate group causing its maxima to shift to lower energy. At 2878, 2930, 2970, 3195, 3242, and 3497 cm^−1^, characteristic bands of aliphatic chains can still be seen despite the profound overlapping band of the gypsum matrix. Similarly, at 1450 cm^−1^ and 1540 cm^−1^ primary and secondary amine peaks from both NGGs are detectable but with much less intensity than the raw gels. The sulfate band (1270 cm^−1^) is very strong and hinders the detection of any changes occurring to NGGs in that region. Increased intensity in *ν*(C=O) stretching frequency band centred at 1793 cm^−1^ as well as the energy band 2500–2600 cm^−1^ is observed for both applied NGGs. The possibility for this band to be an overtone for the band at 1600–1750 cm^−1^ is unlikely since the signal is enhanced in the difference spectra. Therefore, it is more likely to be assigned to *ν*(S–H) stretching frequency which suggests a chemical bonding between interface's sulfates and the stabilising ligand in the NGG. LCF of XANES spectra (ESI, Fig. S7†) shows good agreement with DRIFT results.

NGG stabilisers exhibit strong *ν*(N–H) bending frequency bands at 1630 cm^−1^ and 1660 cm^−1^ which are hindered under clay–gypsum main doublet band 1550–1750 cm^−1^. Difference spectra of NGG-1b provide a positive signal indicating the presence of NGG-1b after application. Two more stabiliser bands at 2850 and 2950 cm^−1^ assigned to *ν*(C–H) stretching of aliphatic hydrocarbon chains are also present and unchanged. Similarly, is the *ν*(N–H) stretching band observed at 3080 cm^−1^. However, the *ν*(O–H) broad vibration band at 3411 cm^−1^ is not seen as in raw NGG spectra indicating a chemical change at the hydroxyl end of both NGG-stabilisers. A broad band at 3411 cm^−1^ is assigned to the *ν*(O–H) vibration, while the N–H stretching band is observed at 3080 cm^−1^. Two bands at 2950 and 2850 cm^−1^ are assigned to *ν*(C–H) and *ν*(N–H) stretching of aliphatic hydrocarbon chains and amine salts of the stabiliser, respectively. At 3640–3700 cm^−1^ and 3800–3950 cm^−1^ new vibrational bands are observed in the difference spectra. Both do not originate from raw support materials or raw NGGs. However, they are commonly assigned to *ν*(O–H) stretching in alcohols.

#### Molecular assessment at the interface with gypsum

At the gypsum interface (Fig. 5l), both NGG-1b and NGG-1c behave similarly and show no detectable spectral change after interaction with the interface. The negative difference means that the support features are prevailing over the NGG. Difference spectra show no features in the positive direction indicating no formation of new bonds, *i.e.* no interaction between applied NGGs and the gypsum interface. At 1450 and 1540 cm^−1^ primary and secondary amine peaks from both NGGs are detectable but with much less intensity than in the raw gels. Similarly, peaks at 1019 cm^−1^ and 1088 cm^—1^ are present. The sulfate band (1270 cm^−1^) is very strong and hinders following up any change occurred to NGGs in that region. No alteration in the peak position of gypsum sulfates is observed, indicating no alteration in chemical bonding. At 2850 and 2950 cm^−1^, bands assigned to *ν*(C–H) stretching of aliphatic hydrocarbon chains can still be seen despite the profound overlapping band of the gypsum matrix. LCF of XANES spectra shows good agreement with DRIFT results (ESI, Fig. S7†).

NGG-1b and NGG-1c interact differently when applied on the four interfaces composing the gilded structure. The results of XANES-LCF for both NGGs at the four interfaces indicate the absence of unfavourable chemical changes occurred to both experimental gold conservation materials NGG-1b and NGG-1c upon application. The NGG-1b interaction with all four interfaces shows superficial agglomeration of nanoparticles mainly photoinduced by incident X-rays. At iron-based interfaces, NGG-1b shows a chemical interaction from the ligand end. This means chemical bonding is in fact taking place besides the commonly known physical bonding between an adhesive and a gold layer. NGG-1c, however, shows a tendency to form ‘temporary’ oxidised species, both photo-induced and interface-induced. These were observed at all interfaces with a higher frequency at gold and gypsum. It is preferable to use a less interactive and more stable conservation material.

The synthesised experimental conservation materials are designed to employ physical re-adhesion between the delaminated gold layer and its support through the stickiness property of gelatin. This was visually assessed upon the application of the gel which showed very good adhesion. No chemical change of bulk gold is detected upon the application of the conservation material as verified by XANES and EXAFS. However, the conservation materials show intermediate charged species where the gelatin interacts with bulk gold, re-distributing the charge density around gold nanoparticles. A schematic illustration showing the results and possible interaction pathways between the NGG and the studied interfaces can be found in ESI Fig. S9.† This, however, did not influence NGG adhesion strength nor its stability after drying.

#### Physical, rheological and optical properties of applied NGGs: some aspects of application parameters

Visual assessment of the intervention carries qualitative value and varies hugely in cases of delamination and deep crack phenomena. Therefore, visual assessment should not be used as the main and only assessment method. Since the 1970s, many researchers focused on theoretical and experimental correlations between rheological properties (viscosity, gelation temperature, and surface tension) and their impact on penetration depth and deposition of various fluids and hydrogels.^43–47^ Numerous studies focusing on binders and adhesives used in cultural heritage conservation have also been investigated since the 80s of the last century.^48,49^ Plesters (1956)^50^ introduced a concrete base for studying a wide range of binders on cross-sections. Potthast (2003)^51^ and Hummert (2013)^52^ suggested fluorescence staining on cross-sections to investigate adhesive penetration and deposition following approaches used in medical applications. Sandu *et al.* (2012)^53^ reviewed staining methods and materials used to characterise organic materials including adhesives and binders of painted and polychrome works of art. However, our approach used simple, available, and non-destructive techniques.

In the following sections, the results of our *in vitro* experiments measuring some aspects of the applied experimental conservation materials (NGG-1b and NGG-1c) that directly influence the conservation intervention are presented. These include lateral distribution on the surface, chromic change, number of applications, and surface tension.

#### Lateral distribution on the surface

When an adhesive material is applied over a surface of the porous material, it spreads laterally and vertically into the surface material. This distribution is affected by adhesive's concentration, viscosity, and application conditions (temperature and humidity). Lateral distribution of the adhesive impacts the adhesion strength. Therefore, it was experimentally measured at the four interfaces to better evaluate the adhesive suitability as a conservation material.

**Fig. 6:**
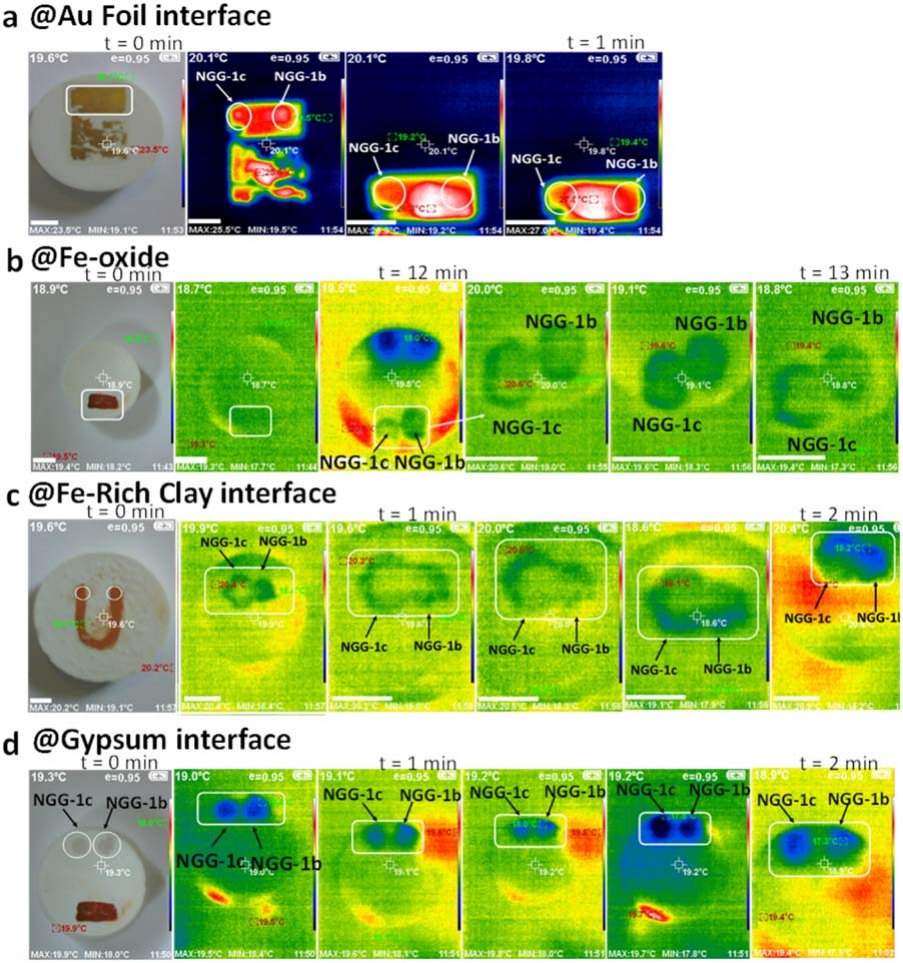
Thermal imaging assessment of *in vitro* application of NGG-1b and NGG-1c over (a) gold foil, (b) iron oxide, (c) iron rich clay, and (d) gypsum interfaces. The scale bar is 1 cm for all images.

The lateral distribution of NGG spread was measured over the four interfaces following the method suggested by Zhang (2018).^54^ Thermal Imaging was used together with vernier caliper measurements (see the ESI† for technical details) to measure lateral distribution of NGG-1b and NGG-1c over the four interfaces. The results are summarized in Table 3 and are shown for each individual interface in Fig. 6. Lateral distribution of applied drops is a dynamic process which means it changes dimension over time (0–13 minutes). This is seen at interfaces with higher porosity (iron based) faster than those with lower porosity (Au foil). Therefore, several sequential thermal images were captured to provide a glimpse on the ‘surface dynamics’ of the applied NGG over time.

**Table 3:** Results of lateral distribution of NGG-1b and NGG-1c applied over the four studied interfaces *in vitro* supported on the gypsum bulk substrate (*the first spread value is over the iron layer and the second is over gypsum. **The first value is for a region with darker NGG color and the second is for a lighter color). Measurements were performed at 23 ± 1 °C and 42 ± 2% relative humidity

Interface	Measurement type and parameters (±SD)		Notes
	H-spread (±0.5 mm)	V-spread (±0.5 mm)	
**NGG-1b interface with**			
Au leaf	—	—	Not visible
Fe_2_O_3_	7, 8*	7, 10*	Symmetric round, then semi-symmetric round over gypsum
Fe-rich clay	6.5	7	Symmetric round
Gypsum	10	10	Symmetric round

**NGG-1c interface with**			
Au leaf	7	7	Symmetric round
Fe_2_O_3_	7, 7*	5, 9*	Symmetric round, then semi-symmetric round
Fe-rich clay	10.5	12	Semi-symmetric round
Gypsum	8, 13**	8, 13**	Symmetric round

As shown in Fig. 6a, the spread of the drop was visible for NGG-1c only and was measured to be 7 × 7 mm ± 0.5 mm in both the *x*-direction and *y*-direction, spreading in a symmetric round form. The spread over the iron oxide interface was better traceable due to the temperature difference between NGGs and the interface. Scale-wise, as seen in Fig. 6b (left to right), the droplet's lateral spread is evaluated from the width of the thermal image of the applied droplet as well as with vernier caliper direct measurements.

It is observed that both NGGs expand initially mostly in a symmetric round form. Then it continues spreading beyond the iron oxide layer reaching the gypsum support where the spread changes into a semi-symmetric round form. Finally, the spread shrinks and appears to be absorbed by the support. Thermally, as supported by the color scale, the surface starts with green thermal color with homogeneous temperature (18.7 °C ± 2 °C), then upon the application of both NGGs, the surface shows relatively a warmer signal. Gradually, the surface temperature changes to 20.6 °C ± 2 °C at the application spot over *ca.* 30 seconds then cools down to 18.8 °C ± 2 °C. However, the principle is valid and new to the conservation field as an assessment method for lateral spread of materials on surfaces. In a similar way, tracing droplets spread over the iron rich clay interface was conducted. Scale-wise, as seen in Fig. 6c (left to right), it is observed for both NGGs that the width of their droplets expands gradually. NGG-1c was spreading in a semi-symmetric round form while NGG-1b spreads in a symmetric round form in both the *x*- and *y*-directions. It is also observed that NGG-1c spreads wider and faster than NGG-1b, and then it continues beyond the clay layer reaching the gypsum support. Finally, the spread shrinks and appears to be absorbed by the support. Thermally, the surface initially has a homogeneous temperature then upon application of both NGGs, the surface shows a warmer signal (20.4 °C ± 2 °C). At the fourth interface of gypsum, as shown in Fig. 6d (left to right), the width of both NGG droplets expands initially and gradually into symmetric round forms. NGG-1c spreads wider and faster than NGG-1b. At the end, the spread shrinks and appears to be absorbed by the support.

#### Number of applications

**Fig. 7:**
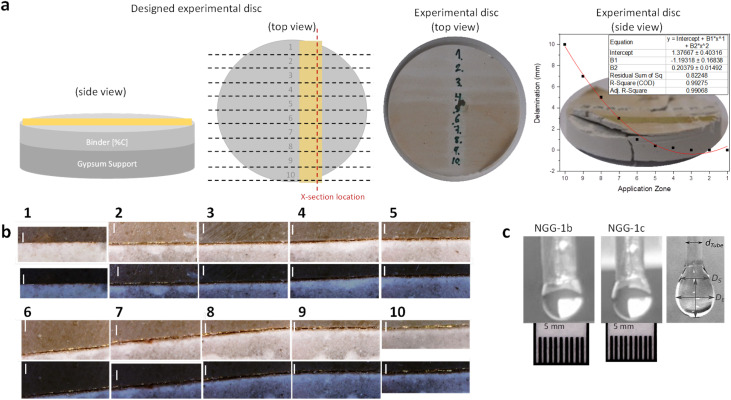
(a) Side and top views of the experimental disc as designed and produced with the impact of the number of applications plotted on the right side. (b) Cross-section images under visible and UV light illumination for the ten application zones as indicated by numbers. The scale bar is 100 μm for all images. (c) (Left and middle) NGG-1b and NGG-1c droplets suspended at the tip of a Pasteur pipette. (Right) Measured diameters used in the equation mentioned in the text.

The total concentration and volume of the adhesive material applied on the surface to be conserved depend on the number of applications of the adhesive. Both the final concentration and volume of the adhesive influence its penetration depth and adhesion strength. Therefore, a number of applications of the adhesive gel were experimentally tested on replica samples. Under room conditions (23 °C and 44% RH for all measured samples), gold foil (200 nm thick) was applied over a gypsum disc (5.1 cm diameter and 1.2 cm thick) using the NGG-1b experimental adhesive. The disc was used for 10 consequent adhesive zones (using a paint brush) starting with one vertical application across the disc at one end of its diameter and ending with 10 applications at the other end, each time increasing one more ‘brush’ application. Upon complete dryness (7 days), visual assessment and a cross-section of the whole disc were made to assess semi-quantitatively the impact of the number of adhesive applications on adhesion strength, adhesive's penetration depth, and adhesive's in-depth distribution. Visual assessment of delamination on the disc and optical microscopy on the prepared cross-section were used to qualitatively assess adhesion strength. Delamination of the upper layer was visible and varied across the ten investigated zones. The delamination scale was measured and is plotted in Fig. 7 (top right). A 2^nd^ order polynomial fit of delamination experimental data was applied and showed very good regression *R*^2^ = 0.99275. The edge of the disc where only the adhesive was applied without gold on top, showed the highest influence of the increased number of applications on adhesion to gypsum. An exponential increase in delamination starts after the 5^th^ application zone and ends at the 10^th^ with approximately 10 mm delamination as directly measured on the disc. At the 4^th^ application zone, a relatively small delamination (200 μm) is observed, while no detectable delamination is observed for the first three application zones.

Assessment of the ten application zones on the cross-section using optical microscopy was then implemented. As shown in Fig. 7b and Table 4, the ten zones are presented under visible and UV illumination. The color of NGG-1b (very light purple) is distinguishable compared to white gypsum. This is visible in the cross-section images. In-depth distribution of NGG-1b appears to be irregular. In the 1^st^ zone, it appears to have been passed through the whole stratigraphy (500 μm). While in the 2^nd^ and 3^rd^ zones, it shows full penetration in the first 70–100 μm followed by deposition in the rest of the gypsum matrix (400 μm). In the 4^th^ to 10^th^ zones, it shows full penetration in the first 50 μm and then deposition in the middle part (50–250 μm) of the full cross-section width. The gold layer also started to get delaminated in these zones. The subsequent application of the adhesive causes an accumulative increase of adhesive concentration which expectedly influences its overall viscosity within the gypsum matrix. Therefore, it is expected to notice shallower penetration depths compared to zones with less viscous adhesive applications (a smaller number of applications). Due to high viscosity, back flow of the adhesive to shallower depths or to spread horizontally is also possible. In summary, it is proved that the number of adhesive applications influences its penetration depth and adhesion strength. The recommended maximum number of applications in this case is three, or similarly, the maximum adhesive concentration is recommended to not exceed 9% wt/wt.

**Table 4:** Results of penetration depth and distribution of NGG-1b applied with multiple applications over the gypsum matrix (−: not observed, +: little, ++: moderate, +++: strong, ++++: v. strong)

Application zone #	Accumulative concentration (wt/wt% NGG-1b)	Delamination of Au foil	Detachment of Au–gypsum	Penetration depth (μm)	Distribution of NGG
1	3%	−	−	500	At the surface and through full stratigraphy
2	6%	−	−	500	
3	9%	−	−	500	
4	12%	−	+	100–250	Deposition in the middle part of the cross-section
5	15%	+	+	100–250	
6	18%	+	++	100–250	
7	21%	+++	+++	100–250	
8	24%	+++	++++	100–250	
9	27%	+++	++++	100–250	
10	30%	+++	++++	100–250	

#### Surface tension

**Fig. 8:**
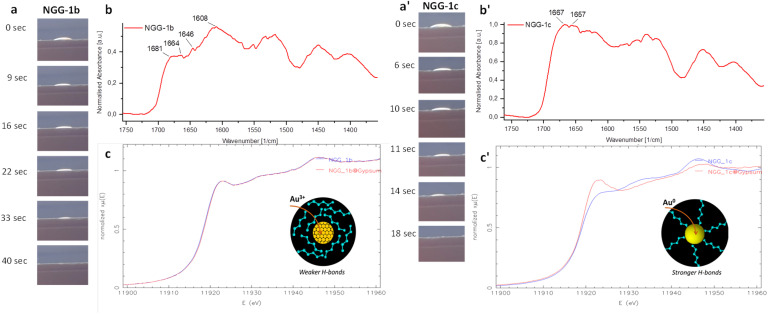
Time-dependent absorption at the interface with gypsum for (a) NGG-1b and (a′) NGG-1c. (b) FTIR spectra of raw NGG-1b and (b′) raw NGG-1c for amino acids region 1350–1750 cm^−1^. (c) Au-L_3_ XANES spectra of NGG-1b and (c′) NGG-1c, raw and over gypsum. Insets in (c and c′), respectively, illustrate schematically the impact of capping orientation on hydration water and charged Au-nanoparticles in NGG-1b, and neutral AuNPs in NGG-1c.

Surface tension was measured using a simple and affordable method that has been experimentally validated and scientifically accepted (see the ESI†).^15^ Measurements were performed on solutions of the experimental nanogold gels NGG-1b and NGG-1c. Surface tension for both were measured under room conditions (23 °C and 44% RH) (Fig. 8).^55^ When a drop is suspended at the tip of a tube, the shape of the drop is described using the local Laplace equation, where the weight of the drop and surface tension forces are balanced:2
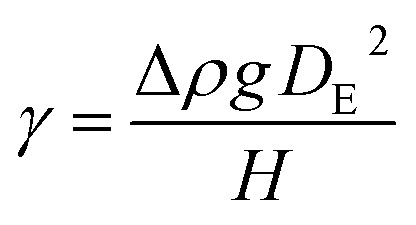
where Δ*ρ* is the density difference between the adhesive and air, *g* is the gravitational constant (*g* = 9.81 m s^−2^), and *D*_E_ is the maximum diameter of the pendant drop. A dimensionless function (1/*H*) of the ratio between *D*_S_ and *D*_E_ (Fig. 7c) is used to explain the specific shape of the drop due to gravity. *D*_S_ is defined as the diameter of the drop at a distance *D*_E_ from the bottom of the drop. The (1/*H*) function can be approximated using a simple analytical formula:3
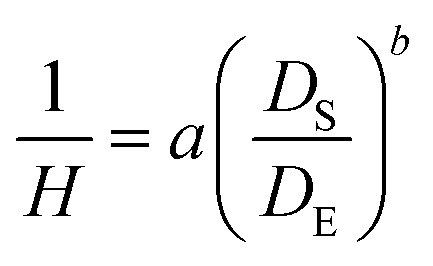
where *a* = 0.345 and *b* = −2.5 according to ref. 56. In reference to Loeb^57^ work, our results indicate that NGG-1b bears a similar surface tension value (*γ*) for 3% gelatin solution, while an increased value for NGG-1c is obtained. However, our measurements were performed at room temperature (23 °C) while Loeb's were obtained at 40 °C. Comparable experimental conditions are needed to obtain a more accurate comparison. Thermal impact on surface tension is neither simple nor a linear relation, it is dependent on the concentration, pH, and temperature of the adhesive.

#### Chromic change

**Fig. 9:**
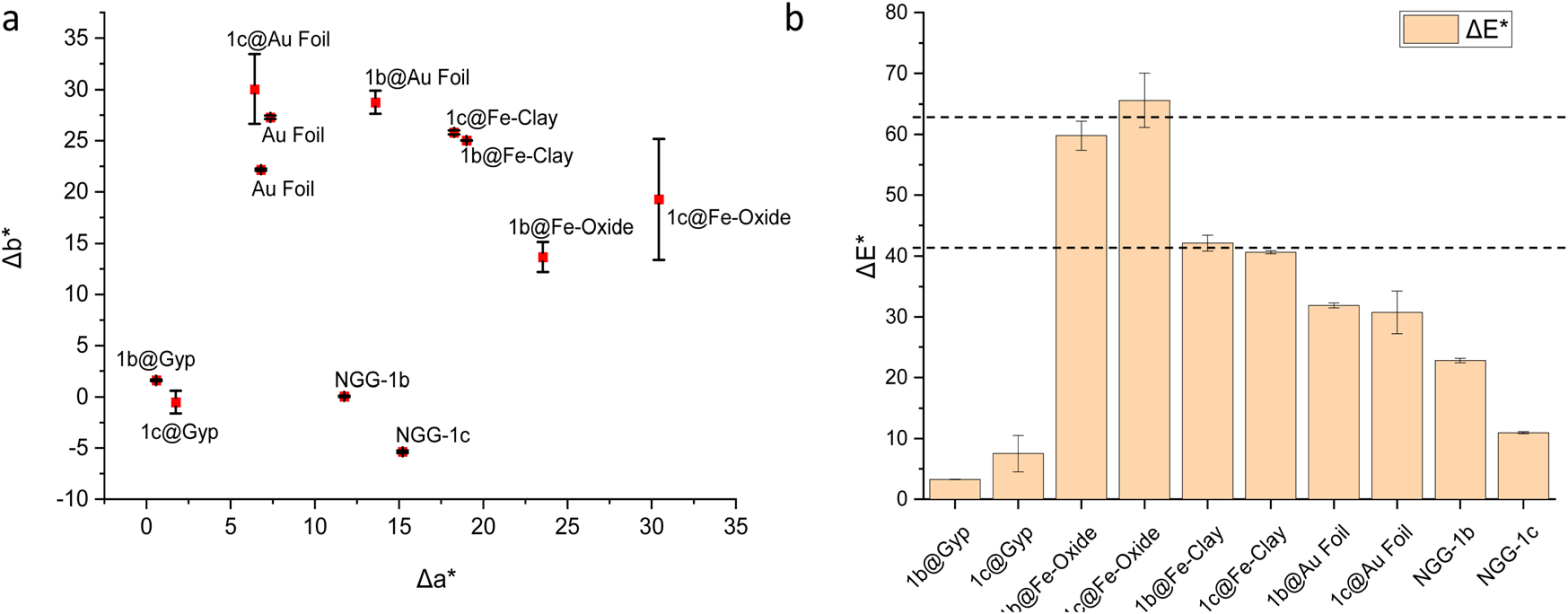
Colorimetric values for NGG-1b and NGG-1c upon application at the four interfaces (a) Δa* and Δb*, and (b) ΔE* with mean values of both iron-based interfaces are shown in horizontal dashed lines.

From a conservation point of view, the application of a light red to light purple material above a darker red iron oxide layer will not influence the final color change adversely. Color coordinates of dry films of all NGGs were recorded at three different positions for each gel. The final visual appearance of the gilded structure after the application of any conservation adhesive gel is important from an aesthetic point of view for the conservation intervention. As the intervention is meant to be underneath gold, the chromic alteration of the bole or paint layer directly under the gold is what is considered here. Therefore, raw NGG-1b and NGG-1c were applied over iron oxide (red) and iron rich clay (orange) interfaces. Applications over gypsum and gold were additionally made for comparison. Colorimetric measurements were performed using a portable colorimeter. Measurements were acquired in reference to the CIE L*a*b* chromaticity diagram.^20^ Each adhesive was tested at three points on the surface and the average was used for plotting the comparative (Fig. 9a and b). It is to be noted that the final visible color is affected by the original color of the interface and adhesive's coverage (lateral spread and in-depth penetration). Color differences Δ*E* were calculated from the collected color coordinates for each measured point using the CIE 1976 equation (Commission Internationale de l'Eclairage) ΔE* = [(ΔL*)^2^ + (Δa*)^2^ + (Δb*)^2^]^1/2^. Chromic alteration is considered acceptable if ΔE* mean value is less than the mean value of the related interface. For the interface of red iron oxide, ΔE* mean value is 63 ± 5, and for the orange iron-rich clay interface it is 41 ± 5. As shown in Fig. 9a, measured points at gypsum and gold interfaces as well as raw NGGs show brighter change ΔL* compared to those applied over iron-based interfaces.

It is expected for both iron-based interfaces which have red and orange colors to have high Δa* (redder), similarly for Δb* where the yellow contribution is dominant. Additionally, measured points at gypsum and gold interfaces as well as both raw NGGs show less change in the red-green scale Δa* compared to those applied over iron-based interfaces (Fig. 9a). The change on the yellow-blue scale Δb* at the gypsum interface is insignificant compared to those applied over iron-based interfaces. Acceptable chromic alteration is seen in ΔE* values. All values are below the mean values of both iron-based interfaces, as shown in Fig. 9b, horizontal lines, which in terms of their final visible color at the surface from a conservation point of view means an aesthetically acceptable intervention. Importantly, a variation in the interface chromic response to the adhesive material is seen in the discrepancy of colorimetric parameters for both NGGs at the same interface.

#### Long term stability of the synthesized nanogold gels

Long-term stability of a material can be tested through accelerating deterioration factors known to cause damage and deterioration for the material in interest, in other words, artificial aging. For Petra's wall paintings and stucco, the main deterioration factors are environmental sources. These include solar radiation, thermal, hygral, and soluble salts. Therefore, an accelerated deterioration environment was designed following an elevated factor of the main cause of deterioration. In this research, two factors were tested. Solar radiation through exposure to UV light of 366 nm (A.KRÜSS Optronic GmbH, UVT3600) for 240 minutes. And thermal deterioration, where samples were exposed to an elevated thermal environment at 100 °C for 180 minutes. A combination of thermal and UV radiation was also tested starting with heating at 100 °C for 180 minutes followed by an exposure to UV 366 nm radiation for 240 minutes. A reference sample was kept for comparing and monitoring changes caused by each deterioration factor.

A few drops from each synthesized NGG were applied over glass slides and left to dry at room temperature and pressure. Upon complete dryness, after 72 hours, NGG dry films were artificially aged under the described conditions above. Another stability test was to follow changes at room temperature and pressure with day light exposure for long time of the gel form of the NGGs. The nanogels (10 drops) were then left in sealed test tubes under laboratory conditions and visual assessment by the naked eye was used to evaluate any change. Time periods were 1 month, 6 months, 1 year, and 5 years. Examination after UV-exposure shows no optical change, no crack development, and no volume change. Examination after thermal aging and combined UV-thermal aging conditions, shows cracking and embrittlement of all NGGs dry films. Examination of the gel-form of all NGGs aged over time under laboratory conditions shows the following results:

• Gelatin-stabilized NGGs (NGG-1a and NGG-1d) changed and started to show clear biological activity after one month. AuNPs started precipitating and separating from the stabilizing gelatin polymer. Over longer time, no further changes were observed.

• Gelatin-stabilized NGGs (NGG-1b and NGG-1c) didn't show any biological activity, but it started precipitating and separating from the gelatin polymer after one month. Over longer time, no further changes were observed.

• Gum arabic-stabilized NGGs and Paraloid B72-stabilised NGGs were stable over the entire examination period and did not show any alteration. Partial separation of AuNPs from the gum was observed but it was reversible upon shaking the vial.

From the conservation point of view, after over five years of natural aging of all *in vitro* samples, the treated samples did not show any color alteration, size change, aging scent, biological activity, or re-detachment of the treated gold layer. This indicates a successful realisation of the designed and synthesised NGGs where they first regenerated gold adhesion to its support, and additionally took the organic binder to a new stabilised form by introducing gold nanoparticles into their structure and function.

The most chemically stable NGG synthesised is NGG-1b. At the molecular level of interaction, NGG-1c showed a more desirable behaviour from conservation point of view where a less surface tension is achieved, shorter intervention time, and temporary charged species at interfaces. However, a proper conservation material needs to be stable in the short term as well as in the long term and NGG-1b has the favour.

*In vitro* experiments measuring some aspects of the applied experimental conservation materials (NGG-1b and NGG-1c) that directly influence the conservation intervention were evaluated. These included lateral distribution on the surface, chromic change, number of applications, and surface tension. For lateral distribution on the surface, the widest spread of both gels is found to be over gypsum. This is followed by spread over iron-based interfaces by NGG-1c and then NGG-1b. Spread over gold for NGG-1c is of the same size as its spread over the iron oxide interface. Thermally, the application of NGGs causes a temporary warmth of the applied area which varies between 0.6 °C and 2.0 °C above its starting temperature before it returns to its original surface temperature. Acceptable chromic alteration is obtained for both NGGs at all interfaces. All ΔE* values are below the mean values of both iron-based interfaces, which in terms of its final visible color at the surface means an aesthetically acceptable intervention from the conservation point of view. However, a variation in the interface chromic response to the adhesive material is seen in the discrepancy of colorimetric parameters for both NGGs at the same interface.

It was proved that the number of adhesive applications influences its penetration depth and adhesion strength. The recommended maximum number of applications was three, or similarly, the maximum adhesive concentration is recommended not to exceed 9% wt/wt. Time-dependent thermal imaging showed longer adsorption time for NGG-1b compared to NGG-1c. This is in good agreement with surface tension results where NGG-1c is found to have higher surface tension compared to NGG-1b. It can be said that the higher the surface tension, the faster the wettability and adsorption are. Molecular assessment suggests that NGG-1b has randomly oriented gelatin around positively charged gold nanoparticles with weaker H-bonding. Meanwhile NGG-1c is seen more with well oriented gelatin around neutral gold nanoparticles with stronger H-bonding.

## Conclusions

Adhesive polymer-stabilised gold nanoparticles as conservation materials for gilded paintings were synthesised, characterised, and presented. Two natural polymers and one synthetic polymer were used as reducing agents and stabilisers for gold nanoparticles. The production of metal nanoparticles using one-pot green synthesis methods is advantageous when compared to chemical methods by being faster, affordable, and environmentally friendly. Various characterisation methods at physical, chemical, and molecular levels were used allowing identification of particle size and shape of AuNPs, as well as the hydrodynamic diameters. The results show that the three polymers remain chemically intact. The formation of AuNPs stabilized by the three polymers did not influence their structure. However, FTIR and XANES analyses imply the need for improving the stage of separation and purification of AuNPs to reduce any unreacted Au(iii) species before using the products as conservation materials. According to the conservation purpose of NGGs, particle size and shape is not expected to influence the adhesion criteria. Therefore, unifying or modifying shape and size of gold nanoparticles is not needed for further development of the NGGs according to current results. The polymer part of the NGG contributes to conservation action by adhering to both the gold layer and the paint layer underneath. Gold nanoparticles enhance the stability of the polymer and introduce mass conservation to the gold layer by counter-balancing the lost gold mass.

The results shown are opening new possibilities utilizing nanomaterials and modified polymers in the conservation field. Our findings do not only benefit the conservation of Nabataean gilded wall paintings but also other gilded contemporary and historical wall paintings. This research presents an exemplary study that is not restricted to market products, especially when what is available does not solve the problem and when unreplaceable heritage materials are to be urgently preserved.

## Data availability

The data supporting this article have been included as part of the ESI.†

## Author contributions

Maram Na'es: conceptualization, methodology, synthesis, analysis, data treatment, figures preparation, writing original draft, review and editing. Lars Lühl: co-performed XANES analysis, review, and editing. Birgit Kanngieβer: funding, review, and editing. All authors have given approval to the final version of the manuscript.

## Conflicts of interest

The authors have no conflicts of interest to declare.

## Supplementary Material

NA-007-D4NA00877D-s001
