# Supplementary material for: A new conservation material for gold in heritage wall paintings: polymer-stabilized nanogold gels (NGGs)
Source: Nanoscale Adv. 2024 Dec 11;7(4):1061–76. doi: 10.1039/d4na00877d (PMC11667577; doi:10.1039/d4na00877d)
Supplement: NA-007-D4NA00877D-s001 [file NA-007-D4NA00877D-s001.pdf]

Supplementary Information (SI) for Nanoscale Advances.
This journal is © The Royal Society of Chemistry 2024

Supporting Information

A new conservation material for gold in heritage wall paintings: polymer-stabilized nanogold gels (NGG)

Maram Na'es,[a] Lars Lühl,[a,b] and Birgit Kanngießer[a]*

[a] Institute for Optics and Atomic Physics, Technical University Berlin, Hardenbergstr. 36, 10623 Berlin

[b] current address Pfeiffer Vacuum GmbH, Germany.

S1. Materials
S2. Synthesis approaches
S3. Characterization methods and settings
- Confocal Micro X-Ray Fluorescence (3D-µXRF)
- XANES and EXAFS
- FTIR
- DLS
- SEM, ESEM, TEM, OM
- Colorimetry, Surface Tension, Thermal Imaging

S4. Analytical results
S4.1 DLS
S4.2 FTIR
S4.3 XANES
S4.3.1 In-vitro assessment: XANES measurements for pure and applied NGGs over the four studied interfaces
S5. Some aspects of application parameters
S5.1 Lateral distribution on the surface
S5.2 Penetration Depth of NGG
S5.3 Colorimetric parameters

S1. Materials

Table S1. Preparation details of reactants and polymers stock solutions used in the experiments

Stock solution	Polymer or reactant	Polymer weight (g) ± 0.01 g	Solvent	Solvent volume (mL) ± 1 mL	Reaction temperature (°C) ± 2 °C	Storage Conditions
3.3 % wt/wt Gelatin solution	Crushed gelatin sheets	3.30	deionized water	100	80	4 °C ± 2 °C
3 % wt/wt Gum Arabic solution	Powder Gum Arabic	3.00	deionized water	100	80	4 °C ± 2 °C
3 % wt/vol Ethyl methacrylate copolymer (Paraloid B72)	Paraloid B72 (in pearls)	3.00	Acetone	100	RT (22 °C)	4 °C ± 2 °C
10 mM $HAuCl_4.3H_2O$ solution	$HAuCl_4.3H_2O$	1.00	deionized water	250	RT (22 °C)	In a well-sealed dark brown glass container wrapped with multiple layers of Al-foil at RT.
2.2 mM $Na_3C_6H_5O_7$ solution	$Na_3C_6H_5O_7$	1.00	Deionized water	250	RT (22 °C)	RT (22 °C)

All glassware used was cleaned in a freshly prepared aqua regia solution ($HCl:HNO_3$, 3:1 v/v) then rinsed repeatedly with deionized water and left to dry at room temperature prior to use.

S2. Synthesis approaches

S2.1 Green synthesis of Nano Gold Gel (NGG)

Self-prepared shell gold from Doppel Gold leaf with gelatin in one approach, and with gum Arabic in another were synthesized using green chemistry. The first approach is mechanochemically using pestle and mortar, and the second is one-pot biochemical synthesis via Au_2O_3 and $HAuCl_4$ chloride precursors.

S2.1.1 Mechanochemical synthesis (NGG-1a, NGG-2a)

A thin film of gelatin was made by pouring 5 mL of the 3.3 % solution in a petri dish and leaving it to dry to hardness. Then minimal wetting of the film surface with deionized water using a brush was done to allow the gold leaf to stick firmly on it. A transferable gold leaf (Doppel Gold, Kremer Pigmente®) was then applied over and fixed by a gilding brush. After 24 hours, the gilded film was manually crushed to smaller parts then grinded in a mortar and pestle till very fine powder was produced. Prior to storing at 4 °C, 5 mL of 3.3% warm gelatin solution were added to redisperse the synthesized NGG-1a. Similarly, the second approach was prepared with gum Arabic solution (3%, 5 mL) instead of gelatin. And the synthesized NGG-2a was also stored at 4 °C after redispersing in 5 mL gum Arabic solution. The produced NGGs were used as synthesized without any purification, and their color retained gold's golden color.

S2.1.2 Biochemical one-step synthesis

S2.1.2.1 via HAuCl4.3H2O (NGG-1b, NGG-2b, NGG-3b)

NGG-1b

AuNPs were synthesized using $HAuCl_4.3H_2O$ as gold precursor and gelatin as a reducing and stabilizing agent. From a freshly prepared gelatin solution of 3.3 % prepared as mentioned above, 5 mL were added to 5 mL (10 mM) $HAuCl_4.3H_2O$. While heating (80 °C), the solution was vigorously and continuously stirred till the

color started turning pink after around 1 hour. The growth of AuNPs continued for 30 minutes. The color change of the reaction solution was observed from yellow to colorless to grey to pink. The solution was left to cool down to room temperature without disturbance. A darker red color appeared after 24 hours reaction time. The synthesized AuNP gel (NGG-1b) was then purified through a double centrifugation process. The first was performed for 10 min at 10,500 rpm. The supernatant was then decanted, and the denser NGG part was immediately dispersed in 0.5 mL gelatin solution. The supernatant was recentrifuged for 20 minutes at 10,500 rpm and the denser NGG part was added to the first dispersed NGG part. This purified part was set for a second and last centrifugation round for 20 minutes at 10,500 rpm. The supernatant was again decanted and disposed, and the NGG part was redispersed in 0.5 mL Gelatin and stored at 4°C in a fridge. Purification of synthesized NGGs was done for increasing the production yield of the synthesis, and for removing unwanted non-gold components particularly those which can interfere adversely with painting materials, e.g. chloride ions and soluble salts.

NGG-2b

In a similar procedure, NGG-2b was synthesized and redispersed using 3 % gum Arabic solution to reduce and stabilize AuNPs instead of gelatin.

NGG-3b

AuNPs were synthesized using $HAuCl_4.3H_2O$ as gold precursor and Paraloid B72 as a reducing and stabilizing agent. From a freshly prepared 3 % Paraloid B72 solution prepared as mentioned above, 5 mL were added to warm 5 mL (10 mM) $HAuCl_4.3H_2O$. Upon addition, layers separation with floating white foamy material is noticed as expected due to Polymer insolubility in aqueous medium. This means that unreacted Paraloid is automatically excluded from gold solution in the reaction container. However, the whole reaction container was kept under vigorous and continuous stirring for 1 hour without heating. The growth of AuNPs continued overnight at room temperature where the reaction solution turned from yellow to white to colorless to light pink. The synthesized AuNP gel (NGG-3b) was then purified through removal of unreacted floating Paraloid polymer, followed by double centrifugation process as described above. The produced NGG-3b was stored at 4°C in a fridge.

2.1.2.2 via Au2O3 (NGG-1c, NGG-2c, NGG-3c)

NGG-1c

AuNPs were synthesized using gold (III) oxide as gold precursor, and gelatin as a reducing and stabilizing agent. 1 mg of gold (III) oxide (Au_2O_3) powder was added to 1 mL of freshly prepared gelatin solution of 3.3 %. The solution was then vigorously stirred and heated up to 80 °C. Color change of the reaction solution was observed from colorless to grey to brown. The growth of AuNPs continued for 30 minutes. The solution was then left to cool down to room temperature without disturbance. A very light red color appeared after 24 hours reaction time. The synthesized AuNP gel (NGG-1c) was used without further purification to preserve colloidal stability of the synthesized gelatin-capped AuNPs. Visibly unreacted Au_2O_3 at the bottom of the vial were not included in any of the following experiments. The NGG was stored at 4°C in a fridge, except when it was sampled for characterization or further experiments which were performed at room temperature.

NGG-2c

In a similar procedure, NGG-2c was synthesized using 3 % gum Arabic solution instead of gelatin as a reducing and stabilizing agent for AuNPs. Specifically, 1 mg of gold (III) oxide (Au_2O_3) powder was added to 1 mL of freshly prepared gum Arabic solution of 3.3 %. After vigorous and continuous stirring at 80 °C for 30 minutes, the solution started changing color from colorless to grey to brown. The growth of AuNPs was continued for another 30 minutes and the solution was left to cool down to room temperature without disturbance. A very light red color appeared after 24 hours reaction time and the synthesized AuNP gel (NGG-2c) was used without further purification, then it was stored at 4°C in a fridge.

NGG-3c

Reducing Au (III) in Au_2O_3 using Paraloid B72 polymer was tested by adding 1 mg of gold oxide to 1 mL of 3 % Paraloid B72 solution and stirring vigorously and continuously for 1 hour. No reaction and no formation of AuNPs was observed even after 72 hours.

S2.2 Chemical synthesis of Nano Gold Gel (NGG)

S2.2.1 Stabilization with gelatin (NGG-1d)

Stabilization of the synthesized Au-NPs via citrate-substitution with gelatin was employed. Principle of the synthesis follows the work of Neupane (2011)[4]. 5 mL of 3.3 % Gelatin were added to 5 mL of the synthesized Au-NPs. The solution was then vigorously stirred and heated up to 80 °C. Substitution of stabilizing agents and resuming AuNPs growth were continued for another 30 minutes under continuous stirring. The solution changed color to purple then was left undisturbed to cool down naturally at room temperature. Afterwards, purification of the synthesized NGG (NGG-1d) was performed using a double centrifugation process as described earlier, then stored at 4 °C.

S2.2.2 Stabilization with Gum Arabic (NGG-2d)

From a freshly prepared 3 % Gum Arabic solution, 5 mL were added to 5 mL of the synthesized Au-NPs. The solution was then vigorously stirred and heated up to 80 °C. Substitution of stabilizing agents was continued for 30 minutes with stirring. The solution changed color to purple then was left to cool down to room temperature without stirring. AuNP gel (NGG-2d) was used after purification following the same double-centrifugation process described earlier. The NGG was stored at 4°C in a fridge, except when it was sampled for characterization or further experiments which were performed at room temperature.

S2.2.3 Stabilization with Paraloid B72 (NGG-3d)

From a freshly prepared Paraloid B72 solution of 3 %, 5 mL were added to 5 mL of the synthesized Au-NPs. The solution was then vigorously and continuously stirred at room temperature. Upon addition, unreacted Paraloid molecules float as white solid foam above NGG solution. The reaction was kept under vigorous and continuous stirring for 30 minutes without heating during which substitution of stabilizing agents was continued and the solution turned purple. The synthesized NGG-3d was then purified through removal of unreacted floating Paraloid polymer, followed by double centrifugation process as described earlier. The NGG was stored at 4°C in a fridge, except when it was sampled for characterization or further experiments which were performed at room temperature.

Description of in-vitro applications is illustrated in **Scheme S1**.

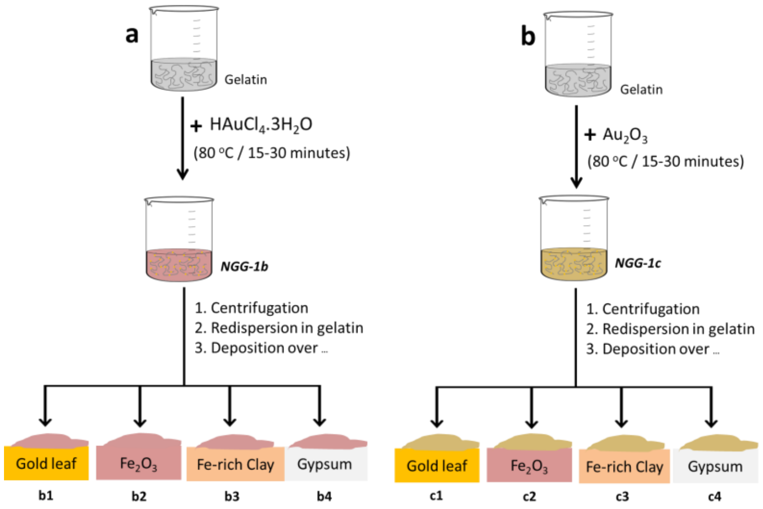

Scheme S1. Illustration of in-vitro samples preparation for (a) NGG-1b and (b) NGG-1c tested at the four interfaces composing the gilded structure.

S3. Characterization methods and settings

Confocal Micro X-Ray Fluorescence (3D-µXRF)

The spectrometer is equipped with a 30 W X-ray tube (Mo target), an energy-dispersive SSD detector, and two X-Ray polycapillary lenses located at 90° geometry with both excitation and detection channels being at 45° with sample surface normal. A full polycapillary lens is mounted in the excitation channel (22 µm focal spot size at 7.5-10 keV), and a half lens in the detection channel. The probing volume is obtained at the intersection of both lenses' foci. Lenses geometry allows both channels to intersect at the focal length of the full lens (manufacturer value: 4.5 ± 0.1 mm), and by adjusting the angles of both lenses a minimal probing volume (maximum intensity) can be approached. The sample is mounted on three directions motorized stage x, y and z and can be controlled by Louvre GUI software. A digital microscope is positioned at the measuring head with a live camera to allow selection of the probing area as well as focusing the microscopic image which allows positioning the probing volume to be at the nearest possible position to the sample surface.

The calibration of the confocal setup includes first calibration of transmission of the excitation lens, and calibration of effective probing volume size and modified integral sensitivity. The integral intensity and depth resolution values for elements of interest found in wall painting materials were obtained by extrapolating the fitted calibration curves. Calibration results are shown in **Figure S1** following the developed calibration procedure for confocal µXRF[5]. Result are listed in **Table S2** and those were used in the quantification of 3D measurements results using XQuant software[5]. The confocal 3D-µXRF setup was also used during allignment stage for µXAS measurements at Bessy II.

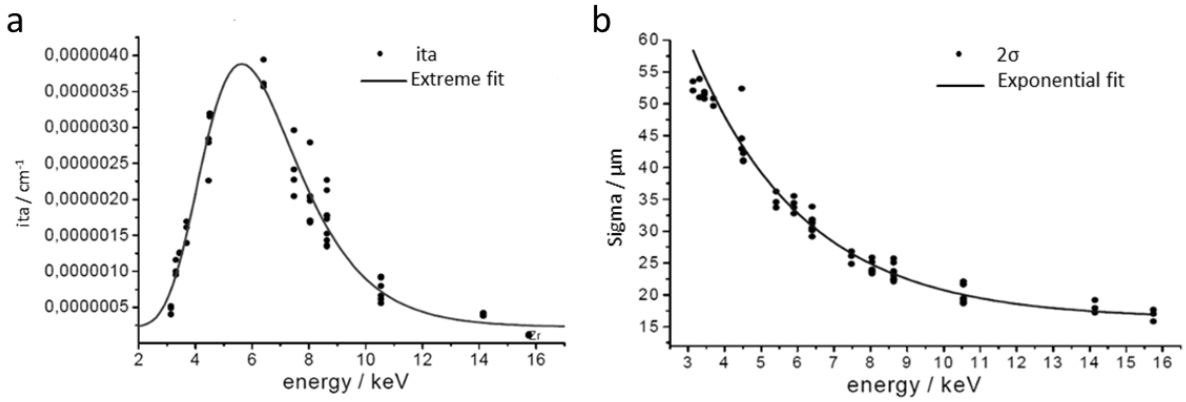

Figure S1. (a) Calibration curve of depth resolution (volume size) fitted with exponential function, (b) calibration curve of integral intensity fitted with extreme function.

Table S2. Integral intensity and depth resolution values of main element of interest to this research

**Fluorescence line**	**energy (keV)**	**2.36.** σ_z **(µm)**	$\acute{\eta}$ **x** E^{-6} **(**cm^{-1}**)**
Ca Ka	3.69	51	1.5
Fe Ka	6.40	31	3.3
Cu La	8.05	25	2.1
Au La	9.71	21	1.0
Hg La	9.99	21	0.92
Pb La	10.55	20	0.72

XANES and EXAFS

XANES and EXAFS measurements were performed at the µSpot beamline of BESSY II[8]. The beamline is located at a 7 T wavelength shifter providing X-ray energies up to 30 keV. The beam is pre-focused by a mirror situated close to the X-ray source and is monochromatized by two pairs of crystal monochromators: Si (111), λ/Δλ~5,000 and a double-crystal mirror with MoB4C coating, λ/Δλ~30 allowing energy resolution of ΔE/E = 1.25 e^{-4}. A pinhole of 100 x 100 μm^2 is placed in front of all samples. The produced flux is monitored with a calibrated ionization chamber. The signal of the ionization chamber is used for normalization of collected spectra. The fluorescence radiation of the sample is detected in reflection mode using a 7-element Silicon Lithium detector positioned at an angle of 90° with respect to the excitation beam. The intensity of the emitted fluorescence radiation is proportional to the absorption. Thus, the obtained excitation spectra are equivalent to conventional absorption spectra. For transmission measurements a second ionisation chamber is placed behind the sample, along the axis of the excitation beam, followed by a 3 µm Au foil and a photodiode. Hence, for each transmission measurement a reference foil is simultaneously measured to check the energy calibration of the monochromator.

Reference XANES spectra were collected in transmission and fluorescence modes for all GoodFellow gold foils with variable thickness (3, 5, 10, 300 µm), pellets of gold oxide Au_2O_3 (20, 10, 5 mg) in cellulose, pellets of gold sulphide Au_2S 20 mg in cellulose, and pellets of Chloroauric acid $HAuCl_4.H_2O$ 30 mg in cellulose. Those include Au foils with variable thickness over $Au_2O_3.2H_2O$. Experimental conservation materials (NGG) were analysed in transmission mode for liquid samples and in fluorescence mode for the dry forms.

Table S3. Scanning procedure used for XANES and EXAFS measurements

Energy Range (eV)	Step size (eV)	# of steps	Acquisition time/step (sec.)	Overall time (sec.)	Overall time (min.)
11800 - 11900	10	10	4	40	0.67
11890 - 11960	0.5	140	4	560	9.33
11959 - 12100	1	141	4	564	9.40
*12000 - 12400	10	40	4	160	2.67
**12098 - 12520	2	211	4	844	14.07
Motors movement time (min.)		30	Approximate overall scan time (min.)		52 (XANES)<br>64 (EXAFS)

* For XANES measurements

** For EXAFS measurements

FTIR

Agilent 4300 handheld Fourier Transform Infrared spectrometer was used. The handheld spectrometer allows non-destructive and non-invasive measurement flexibility in situ. The small (10 x 19 x 35 cm) and lightweight (1.88 Kg + 0.34 Kg batteries), ergonomically designed spectrometer includes three easily changeable sampling interfaces for Diffuse Reflection (DR), Specular Reflection (SR) and Diamond-Attenuated Total Reflection (ATR).[1] For samples with rough surfaces and low reflection (e.g. matrix) the analysis was performed using DR sampling interface, while miniature samples (< 0.5 mm^2) of individual layers were analyzed by ATR. Measurements were performed over the full spectral range 4000-650 cm^{-1} of the built-in DTGS detector. For all interfaces every analyzed sample was collected with 600 scans referenced to 60 background scans unless otherwise mentioned in the text. This is equivalent to 5 minutes acquisition time per sample. The spectrometer has a ZnSe beam splitter and a Michelson interferometer with 4 cm^{-1} maximum spectral resolution. Pre-acquisition mathematical operations of 4 Zero Filling Factor and a HapGenzel apodization were set to all spectra. Background spectra were collected for air in the case of ATR measurements and for KBr or sample matrix for DR and SR measurements.

DLS

Dynamic light scattering (DLS) measurements were carried out on a static/dynamic compact goniometer (SLS/DLS-5000), ALV, Langen, Germany. A He–Ne laser with 22 mW was used as the incident beam. One type of measurements, a dynamic light scattering measurement, in the scattering angles between 30° and 100° were

[1] Last accessed 01 December 2019: https://www.agilent.com/cs/library/flyers/public/5991-4091EN_Flyer_4300_DTGS.pdf

conducted with a step size of 5°. Measuring time at each angle was 45 s. The temperature of the sample was regulated to 293.1 ± 0.1 °K. Liquid samples of the experimental conservation material were diluted and measured.

SEM

For SEM-EDX analysis, samples were carbon-coated beforehand SEM micrographs were taken using a JEOL (Tokyo, Japan) JSM-840 (secondary and backscattered electron detection) with a LINK AN 10000 microanalyser. The setup is equipped with an IR camera (micrographs size: 4096 x 4096 pixel), SAMx EDX-System with Si(Li) detector, X-Ray XFlash-SDD-detector, iMOXS µXRF spectrometer and a XIA LLC digital pulse processor. Micrographs were acquired in a backscattered mode and EDX analysis was performed with an acceleration voltage of 20 keV and a beam current of 1 – 3 x 10−4 mA for 100 s per analyzed point or area.

ESEM

Microimaging under low controlled pressure using an Uwe Binninger Analytik ESEM was performed directly on the samples without any prior preparation. The Microscope is equipped with a Hitachi Tabletop Microscope TM-1000 with magnification from x20 to x10k and operating at 15 keV voltages which provides an imaging of the samples' surface based on the detection of its backscattered electrons.

TEM

TEM measurements were performed to determine particle size and shape of the synthesized NGGs using a Jeol JEM 2200-FS, operating at 200 kV. Samples were prepared by immersing type S-160-3 grids (Plano GmbH) in 0.5 mL volume of NGG solutions. The solvent was evaporated in a dust protected atmosphere under room temperature and pressure. Particle size distributions were obtained by analysing the TEM images using ImageJ software by considering more than 200 particles.

OM

Samples were imaged at ZELMI center at TU Berlin with a Nikon SMZ-U Stereomicroscope and magnifications between x7.5 – x75 using high-resolution digital DS-5MC Nikon camera. A portable USB light microscope was also used at both optical magnifications x50 and x200.

Colorimetry

Colorimetry is the quantitative measurement of the reflection properties as a function of wavelength. Color coordinates were measured to evaluate the visual (aesthetic) impact of the applied NGG adhesive. Colorimetric measurements were performed using a palm size portable 3nh Precision Colorimeter, Model NR10QC. Measurements were acquired in reference to CIE L*a*b*[15] chromaticity diagram and using the color chart standards of B.I.G.[2]. The spectro-colorimeter setup allows an 8° illumination angle and a diffuse reflection component through a measuring aperture of φ 4 mm and φ 8 mm. The measured area is illuminated with the standard illuminant D65 LED blue light and reflected light is detected with a Photoelectric Diode. Color differences ΔE were calculated from the collected color coordinates for each measured point using the CIE 1976 (Commission Internationale de l'Eclairage) equation:

$$\Delta E^* = [(\Delta L^*)^2 + (\Delta a^*)^2 + (\Delta b^*)^2]^{1/2} \quad \text{(S1)}$$

[2] https://www.fotokoch.de/B-I-G-Stufengraukeil-Farbkarte-13cm-18cm_45586.html

Each sample was tested in three points and an average is used for plotting the comparative charts. The color coordinates used L*a*b* represent, respectively: L* the lightness of the color with a minimum of 0=black and a maximum of 100=white, a* position on the red-green axis with negative values=green and positive values=red, b*position on the yellow-blue axis with negative values=blue and positive values=yellow.

Surface Tension

Surface tension measurements were measured following a simple and affordable method which has been experimentally validated and scientifically accepted[14]. A replacement of the proposed smartphone camera, a professional Canon G10 camera was used. Images were treated using their original scale Measurements were performed on solutions of the experimental nanogold conservation materials. Surface tension for both NGG-1b and NGG-1c were measured at room conditions (23°C and 44 %RH) following droplet's simple and affordable method that is experimentally validated and scientifically accepted.[30]

Thermal Imaging

Thermal Imaging was performed using a Htti HT-A1 (V2.1.15) Handheld IR Thermal Imaging Camera. It was used with a matt emissivity of e=0.95 for imaging painted plaster and stucco samples, in-situ and ex-situ. The infrared camera has a resolution of 220x160 pixel, and operates in the range -20 °C – 300 °C with an accuracy of ± 2% equivalent to ± 2 °C.

S4. Analytical results

S4.1 DLS

DLS analysis of two samples (NGG-1d and NGG-3d) was performed[3] to extract information about the hydrodynamic diameter and to complement SEM and TEM findings. Results indicate the formation of spherical particles for both NGGs. Fitting of experimental data was done using the 'Stretched Model' (Figure S2a,b) for NGG-1d and the 'simple Model' (Figure S2 d,e)[4] for NGG-3d, respectively.

Fitting results of NGG-1d data resulted in an average hydrodynamic radius (R_h) of 200 nm ± 4 nm. The use of bulky polymers like gelatin and gum Arabic will increase it. Keeping in mind that the hydrodynamic diameter is dependent on the molecular weight of the stabilizing agent, its concentration, and its size. Nevertheless, agglomeration of AuNPs over time is anticipated and can also lead to larger particle size and spherical shape when examined with DLS. The average particle size of NGG-1d obtained from TEM is 27 nm ± 2 nm. This leads to a rough estimation of capping gel thickness, assuming spherical core particle shape, to be 186.5 nm ± 5 nm (Figure S2c).

Fitting results of the second sample NGG-3d gave an average hydrodynamic radius (R_h) of 30.34 nm ± 0.20 nm. Revisiting size distribution chart of NGG-3d from TEM results shows an average size of 27 nm ± 2 nm and abundance of smaller particle size of 10 and 20 nm. It is then calculated from both average particle size and the hydrodynamic radius, that an estimation of the thickness of capping Paraloid polymer, assuming spherical core

[3] Only two NGGs were examined with DLS. It would have been ideal to analyse all samples equivalently, but DLS became available to us at a later stage of the research where it was used only for selected NGGs.

[4] Fitting was done using an in-house developed program at the research group of Prof. Regine von Klitzing, Chemistry Department, TU-Berlin.

particle shape, to be 16.84 nm ± 1.2 nm (Figure S2f). It is to be noted that agglomeration of NGG due to time difference between synthesis time and analysis time may have contributed hugely to the ‘spherical’ shape results obtained by DLS for both NGGs.

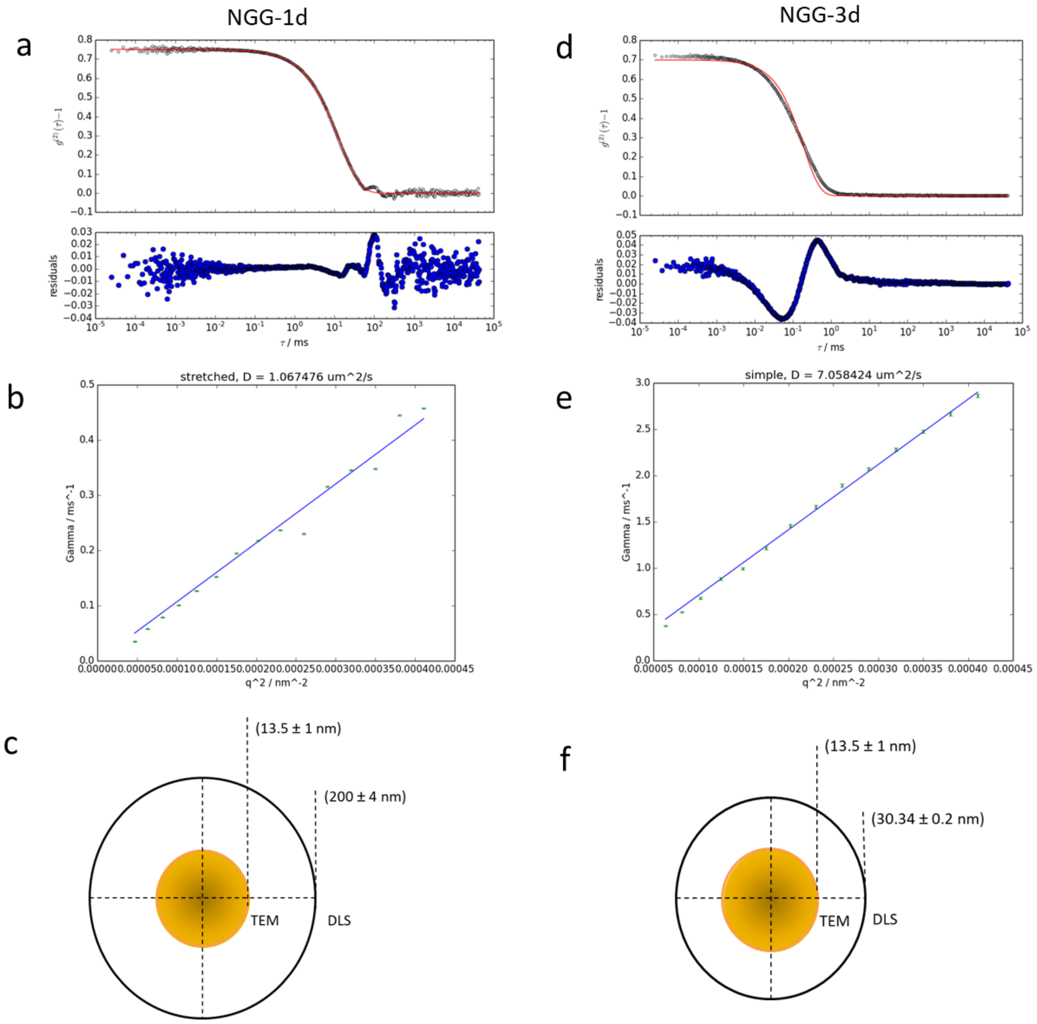

Figure S2. (a) DLS analysis of NGG-1d indicating its large particle size seen in the longer time delay. (b) Fitting of experimental data using the Stretched Model. (c) Illustration of extracted results from TEM and DLS for NGG-1d. (d) DLS analysis of NGG-3d. (e) Fitting of experimental data using the Simple Model. (f) Illustration of extracted results from TEM and DLS for NGG-3d. The hydrodynamic radius (R_h) is also shown.

S4.2 FTIR

Pure gelatin has a strong stretching frequency band of amide I C=O at 1650 cm^{-1}. Amide II N-H bending bands appear at 1545 cm^{-1}. Amide III bands of N-H in-plane bending coupling to C-N stretching vibrations appear at 1243 cm^{-1}. Symmetrical stretching of carboxylate group COO^{-} is seen from 1400 to 1500 cm^{-1}. A broad band at 3411 cm^{-1} is assigned to the O-H vibration, while N-H stretching band is observed at 3080 cm^{-1}. Two bands at 2950 and 2850 cm^{-1} are assigned to C-H stretching of aliphatic hydrocarbon chains. Gum Arabic bands are presented in **Table S4**.

Table S4. Gum Arabic main frequency bands and their assignments [9]

Band Frequency (cm^{-1})	Band Assignment
1049 and 1413	C–O stretch
1612	C–O stretch and N–H bending
3310–3350	N–H stretch for secondary amine
3000–3600	O–H stretch
1550-1640	N–H in primary amines (bending)
3100-3500	N–H in primary amines (stretching)

S4.3 XANES

Table S5: Linear Combination Fit of all synthesised NGGs

Sample	R-factor	$\chi^2(k)$	Reduced $\chi^2(k)$	Au Foil (±SD)	Au_2O_3 (±SD)	$HAuCl_4$ (±SD)	Energy Fitting Range	# of fitted points
**NGG-1a**	0.000080	0.00533	0.0000464	0.982(0.000)	0.023(0.000)	0.000(0.000)	-20 – 40 eV	120
**NGG-1b**	0.000246	0.01757	0.0001502	0.865(0.011)	0.152(0.011)	0.000(0.016)	-20 – 40 eV	120
**NGG-1c**	0.000115	0.00769	0.0000646	0.981(0.005)	0.022(0.004)	0.000(0.006)	-20 – 40 eV	120
**NGG-1d**	0.000137	0.00930	0.0000795	0.604(0.015)	0.109(0.006)	0.286(0.017)	-20 – 40 eV	121
**NGG-2a**	0.000337	0.02243	0.0001950	1.000(0.000)	0.005(0.000)	0.000(0.000)	-20 – 40 eV	119
**NGG-2b**	0.000087	0.00586	0.0000505	0.945(0.013)	0.016(0.005)	0.038(0.014)	-20 – 40 eV	120
**NGG-2c**	0.000082	0.00554	0.0000474	0.801(0.000)	0.000(0.000)	0.199(0.000)	-20 – 40 eV	121
**NGG-2d**	0.000148	0.00989	0.0000853	0.656(0.016)	0.082(0.010)	0.261(0.019)	-20 – 40 eV	121
**NGG-3b**	0.000023	0.00156	0.0000134	0.742(0.006)	0.040(0.003)	0.218(0.007)	-20 – 40 eV	121
**NGG-3d**	0.000207	0.01402	0.0001209	0.755(0.019)	0.055(0.010)	0.191(0.022)	-20 – 40 eV	121

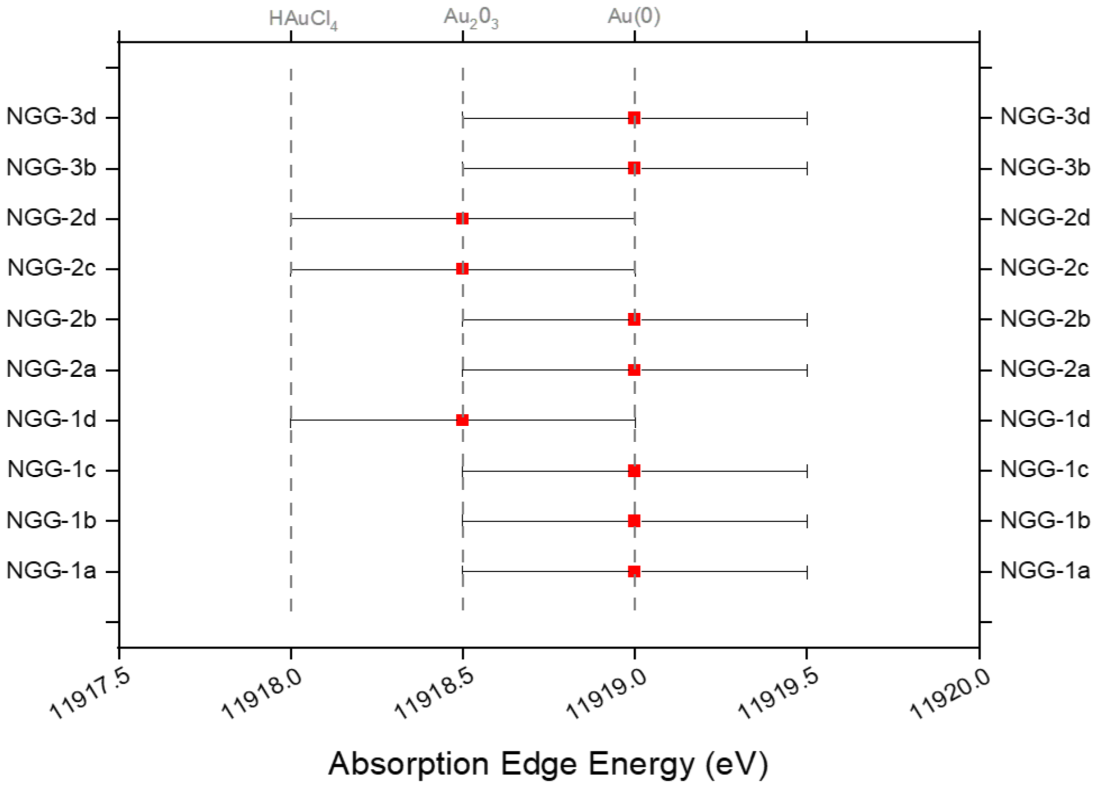

Figure S3: Comparison of absorption edge energies for all synthesized NGGs with Au (0), Au (III) oxide, and Au (III) chloride.

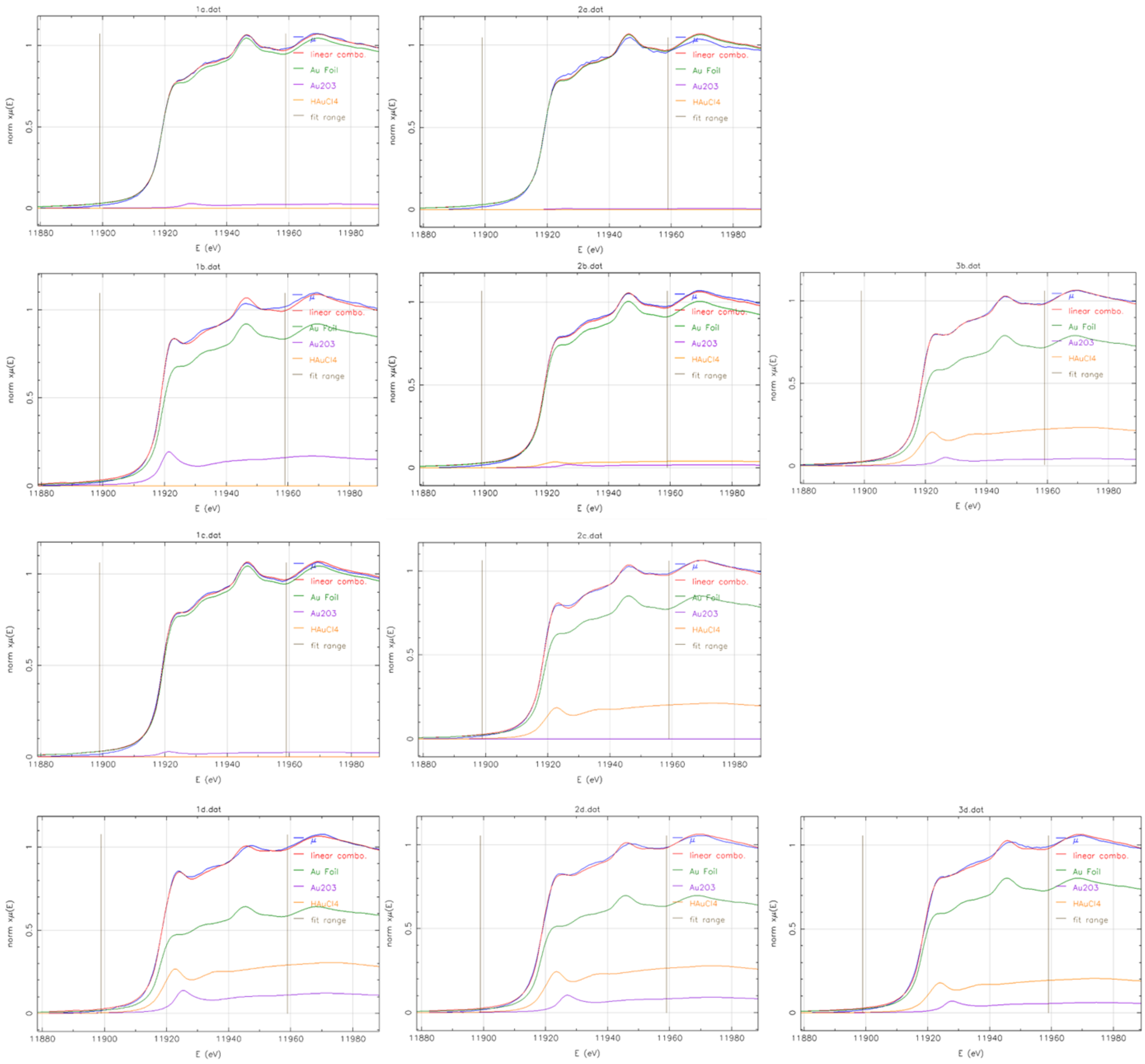

Figure S4: Linear Combination Fit of all synthesised NGGs

S4.3.1 In-vitro assessment: XANES measurements for pure and applied NGGs over the four studied interfaces

Table S6: Absorption edge E_0, edge energy shift, white line intensity, and measurement collection mode of NGG-1b and NGG-1c as dry films of pure gel and of gel applied at the four studied interfaces. Gold standards are also listed for comparison.

Analysed system	E_0 (± SD) (eV)	ΔE_0 (eV) (± 0.5) *	WL Intensity (%)**	Measurement Mode
NGG-1b in vitro				
NGG-1b (gel)	11919.0 (± 0.5)	0	91.2	Fluorescence + Transmission
b1: NGG-1b@Au	11918.5 (± 0.5)	-0.5	83.6	Fluorescence
b2: NGG-1b@Fe-oxide	11918.5 (± 0.5)	-0.5	87.5	Fluorescence
b3: NGG-1b@Fe-clay	11918.7 (± 0.5)	-0.3	85.5	Fluorescence
b4: NGG-1b@Gypsum	11919.0 (± 0.5)	0	91.2	Fluorescence
NGG-1c in vitro				
NGG-1c (gel)	11919.0 (± 0.5)	0	79.4	Fluorescence + Transmission
c1: NGG-1c@Au	11919.0 (± 0.5)	0	84.1	Fluorescence
c2: NGG-1c@Fe-oxide	11919.0 (± 0.5)	0	82.4	Fluorescence
c3: NGG-1c@Fe-clay	11919.0 (± 0.5)	0	83.5	Fluorescence
c4: NGG-1c@Gypsum	11919.5 (± 0.5)	+0.5	89.6	Fluorescence
Au Reference Materials				
Au(0)	11919.0 (± 0.5)		78.5	Fluorescence + Transmission
Au(III)oxide	11918.5 (± 0.5)		127.3	Fluorescence + Transmission
Au(III) chloride	11918.0 (± 0.5)		91.6	Fluorescence + Transmission
Au(I) sulfide	11921.3(± 0.5)		63.9	Fluorescence + Transmission

* In reference to E_0 of the source gel

** WL Intensity: XANES White Line Intensity

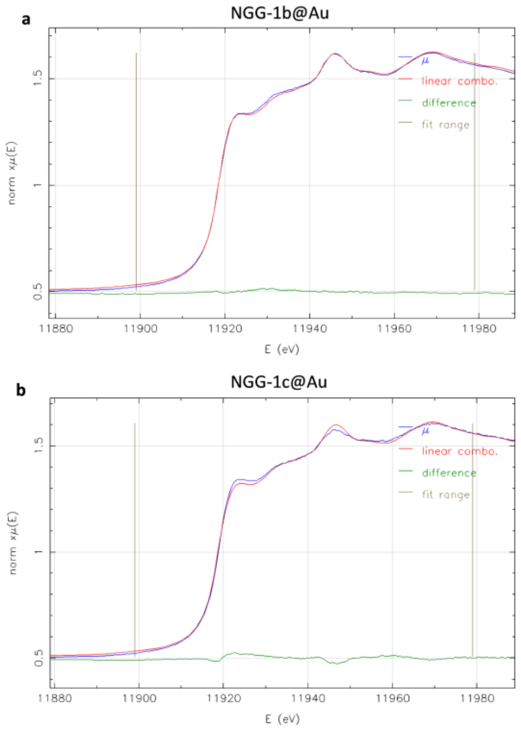

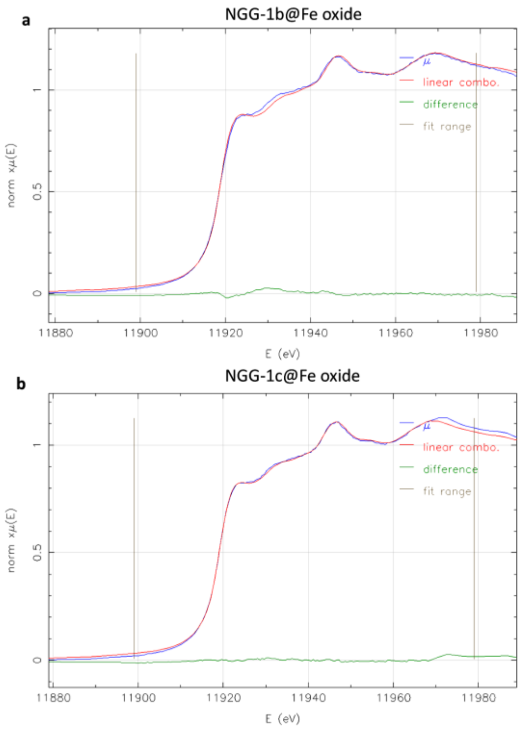

Figure S5: LCF of XANES data at Au L_3- edge for (a) NGG-1b and (b) NGG-1c applied at gold interface. Fitting settings are provided in the text.

Figure S6: LCF of XANES data at Au L_3- edge for (a) NGG-1b and (b) NGG-1c applied at iron oxide interface. Fitting settings are provided in the text.

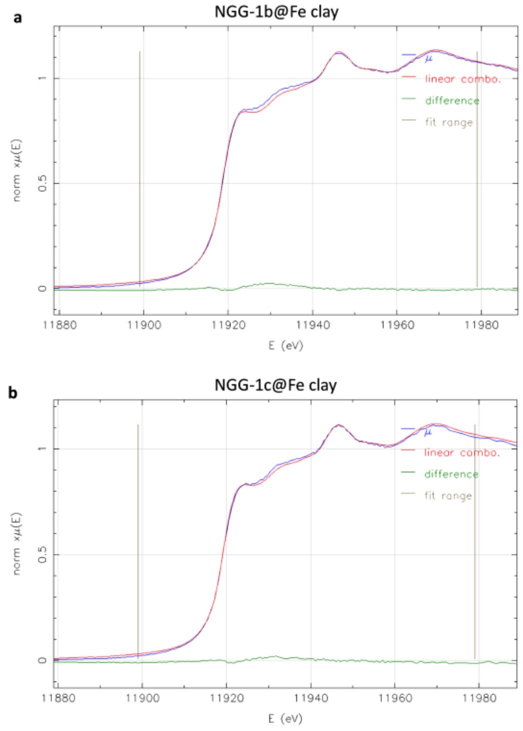

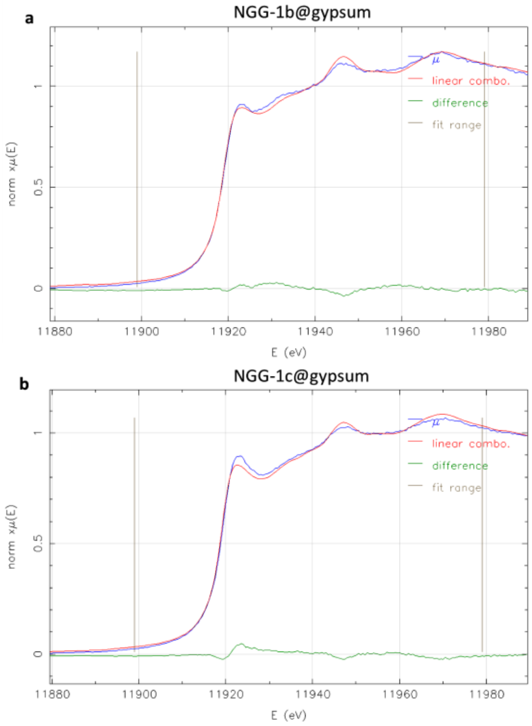

Figure S7: LCF of XANES data at Au L_3- edge for (a) NGG-1b and (b) NGG-1c applied at iron rich clay interfcae. Fitting settings are provided in the text.

Figure S8: LCF of XANES data at Au L_3- edge for (a) NGG-1b and (b) NGG-1c applied at gypsum interface. Fitting settings are provided in the text.

Table S7. LCF results of NGG-1b and NGG-1c applied at the four studied interfaces. Every sample was measured at three different positions and each LCF result represents an average of fitting trials for the three samples spectra. All fitting combinations were applied and the best fit with highest R-factor was used. Raw spectra of NGG-1b and NGG-1c **were not included** in the fitting.

Sample ID	Chemical composition (wt. fraction ± SD) Au(0)	Au_2O_3	Au_2S	R-factor (x 10^{-4})	$\chi^2(k)$	Reduced $\chi^2(k)$	# of fitted points
NGG-1b in vitro	0.938(0.009)	0.055(0.008)	0.000(0.012)	1.64	0.02180	0.0001379	161
b1: NGG-1b @Au	0.990(0.009)	0.050(0.030)	0.000(0.000)	0.58	0.00570	0.0000416	139
b2: NGG-1b @Fe-oxide	0.990(0.000)	0.017(0.000)	0.000(0.000)	1.04	0.01410	0.0000892	160
b3: NGG-1b @Fe-clay	0.990(0.000)	0.013(0.000)	0.000(0.000)	1.06	0.01323	0.0000837	160
b4: NGG-1b @Gypsum	0.980(0.011)	0.020(0.011)	0.000(0.015)	1.98	0.02609	0.0001652	160
NGG-1c in vitro	0.990(0.000)	0.010(0.000)	0.000(0.000)	1.64	0.02180	0.0001379	161
c1: NGG-1c @Au	0.97(0.009)	0.030(0.009)	0.000(0.013)	1.33	0.01592	0.0001008	160
c2: NGG-1c @Fe-oxide	0.980(0.000)	0.015(0.000)	0.000(0.000)	0.67	0.00673	0.0000474	145
c3: NGG-1c @Fe-clay	0.980(0.000)	0.020(0.000)	0.000(0.000)	1.00	0.00974	0.0000711	139
c4: NGG-1c @Gypsum	0.950(0.013)	0.048(0.013)	0.000(0.018)	2.82	0.03167	0.0002004	160

Table S8: LCF results of NGG-1b and NGG-1c applied at the four studied interfaces. Every sample was measured at three different positions and each LCF result represents an average of fitting trials for the three samples spectra. All fitting combinations were applied and the best fit with highest R-factor was used. Raw spectra of NGG-1b and NGG-1c **were included** in the fitting.

Sample ID	Chemical composition (wt %)±SD NGG-1b	Au(0)	Σ*Au(0)*	Au_2O_3	Au_2S	R-factor	$\chi^2(k)$	Reduced $\chi^2(k)$	# of fitted points
NGG-1b in vitro									
b1: NGG-1b @Au	0.178(0.005)	0.862(0.005)	1.040	0.000(0.030)	0.000(0.000)	0.000027	0.00204	0.0000180	118
b2: NGG-1b @Fe-oxide	0.224(0.015)	0.864(0.017)	1.088	0.000(0.023)	0.000(0.000)	0.000021	0.00183	0.0000156	120
b3: NGG-1b @Fe-clay	0.239(0.022)	0.806(0.025)	1.045	0.000(0.033)	0.000(0.000)	0.000046	0.00382	0.0000327	120
b4: NGG-1b @Gypsum	0.994(0.000)	0.000(0.000)	0.994	0.005(0.000)	0.001(0.000)	0.000009	0.00072	0.0000063	120
NGG-1c in vitro									
c1: NGG-1c @Au	0.776(0.013)	0.108(0.011)	0.884	0.152(0.005)	0.000(0.000)	0.000030	0.00230	0.0000200	120
c2: NGG-1c @Fe-oxide	0.739(0.000)	0.267(0.000)	1.006	0.028(0.000)	0.000(0.000)	0.000012	0.00094	0.0000083	120
c3: NGG-1c @Fe-clay	0.822(0.000)	0.208(0.000)	1.030	0.012(0.000)	0.000(0.000)	0.000015	0.00114	0.0000103	118
c4: NGG-1c @Gypsum	0.616(0.000)	0.082(0.000)	0.698	0.305(0.000)	0.000(0.000)	0.000056	0.00405	0.0000356	120

The synthesised experimental conservation materials are designed to employ physical re-adhesion between delaminated gold layer and its support through the stickiness property of gelatin. This was visually assessed upon the application of the gel which showed very good adhesion. No chemical change of bulk gold is detected upon the application of the conservation material as verified by XANES and EXAFS. However, the conservation materials do show intermediate charged species where the gelatin is interacting with bulk gold and re-distributing the charge density around gold nanoparticles, see **Figure S9**. This however did not influence NGG adhesion strength nor its stability after dryness.

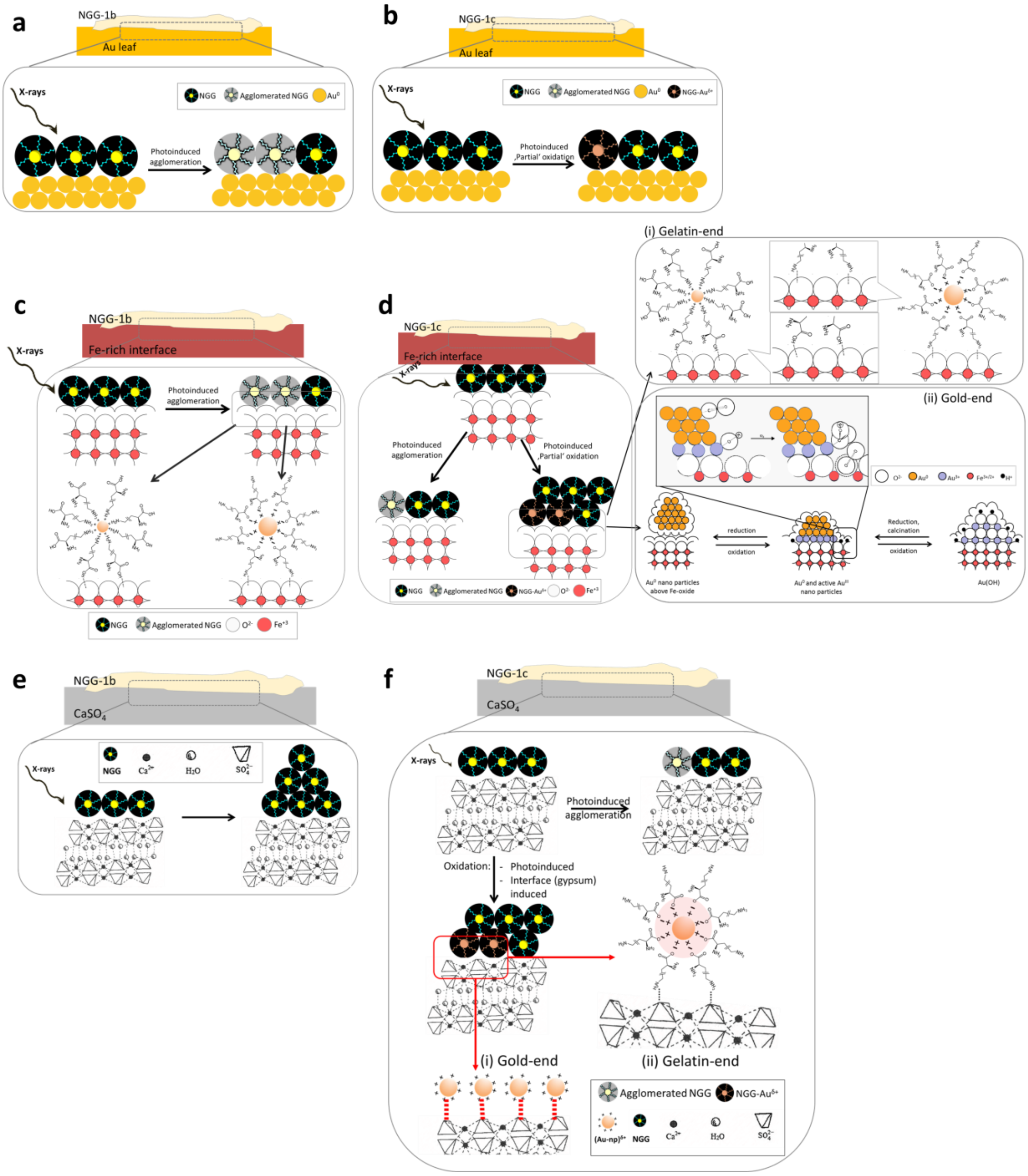

Figure S9: Schematic illustration of NGG-1b and NGG-1c interaction at the interface of (a,b) gold, (c,d) iron-based layers, and (e,f) gypsum, respectively.

S5. Some aspects of application parameters

S.5.1 Lateral distribution on the surface

Thermal Imaging (Hti, HT-A1, Image resolution 220 x 160 pixels, Temperature range -20 °C – 300 °C, accuracy ± 2 % or ± 2 °C) was used together with Vernier Caliper measurements to measure lateral distribution of NGG-1b and NGG-1c over the four studied interfaces (**Figure S10**). This is shown at each individual interface. It is important to highlight the impact of imaging angle on the final image. While the angle is recommended to remain perpendicular to sample surface, i.e. camera's plane and sample's plane remains parallel, it is practically difficult to assure the stability of this factor through all images using free-hand imaging method. Therefore, the camera was fixed on a tripod (tested for verticality using the bubble balance) and the sample was placed over a flat surface. The distance between sample and camera was fixed for all images. However, when reflection was very high the camera was moved nearer to the sample. Imaging was performed at room temperature and pressure. Imaging location didn't include direct sunlight. Artificial light was ca. 2 m away from ceiling level to sample surface, and no heat source was in the vicinity of the imaging location. All images were taken at the same hour (15 minutes in total) and performed under same conditions. These conditions were considered to strengthen consistency of surrounding conditions, and to minimise external thermal contribution to all images.

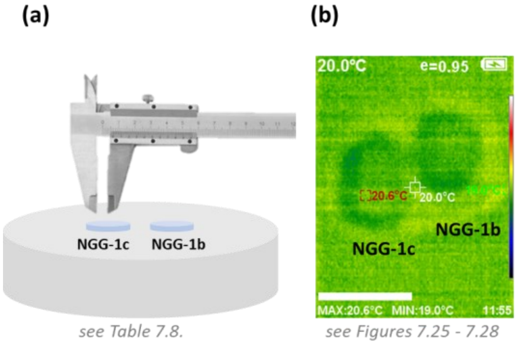

Figure S10: Assessment methods of *in-vitro* lateral distribution spread of applied NGGs. (a) Direct measurement of the maximum spread through its stain using Vernier Caliper. (b) Dynamic Thermal Imaging over time. Scale bar is 1 cm.

S5.2 Penetration Depth of NGG

Since the experimental adhesive NGG-1b contains gold, fluorescence lines of gold in µXRF analysis, both 2D on the cross section and 3D directly on the disc, were used to assess in-depth distribution and penetration depth, respectively. 3D-µXRF was performed on four applications zones (1st, 4th, 7th, and 10th) with depth scans using the following settings: X (1 µm) x Y (1µm) x Z (30 µm), 5 µm step size, 40 steps, 60 second/step. Three scans per application zone were performed and the average of Au-L_α fluorescence line was then fitted with a Gaussian function. The FWHM at every zone was used to indicate penetration depth of NGG-1b adhesive (**Figure S11**). Penetration depths were found to be 19.69 ± 0.24 µm, 20.10 ± 0.23 µm, 17.99 ± 0.32 µm, and 17.94 ± 0.24 µm for the 1st, 4th, 7th, and 10th application zones, respectively. Although this shows that penetration depth of NGG-1b adhesive is not changing with increasing number of applications, two important parameters need to be considered before considering this result correct. First is the technical limitation of confocal 3D-µXRF, where at Au-L_α line (9.71 keV) the depth resolution (2.36 x σ_z) as extrapolated from its fitted calibration curve (**Figure S12** and Table S9) is 21 µm which is the same range found for all four investigated zones. Second is the mass deposition of gold nanoparticles in NGG-1b compared to that of the gold layer at the surface (200 nm thick). The latter has a dominant high intensity compared to nanogold particles that are beneath in stratigraphy. Therefore, their relative intensity will be very weak and unlikely detectable. Based on the discussion above, it is more accurate to assess the penetration depth qualitatively based on microscopic investigations on the cross section rather than quantitatively on confocal measurements.

Table S9: Integral intensity ($\acute{\eta}$) and depth resolution (2.36 x σ_z) values of main elements of interest

**Fl. line**	**E (keV)**	**2.36 x σ_z (µm)**	**$\acute{\eta}$ x E^{-6} (cm^{-1})**
Ca Ka	3.69	51	1.5
Fe Ka	6.40	31	3.3
Au La	9.71	21	1.0

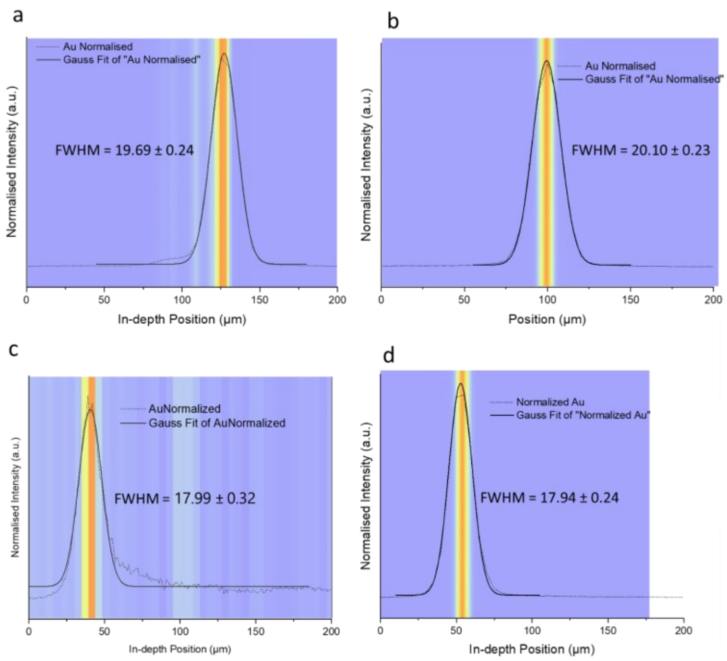

Figure S11: Au-L_α depth profiles of NGG-1b applied over gypsum for (a) 1st, (b) 4th, (c) 7th, and (d) 10th application zones. Presented depth profiles are the average of 3 scans/zone. Measurement settings for all scans: X (1 µm) x Y (1µm) x Z (30 µm), 5 µm step size, 40 steps, 60 sec/step.

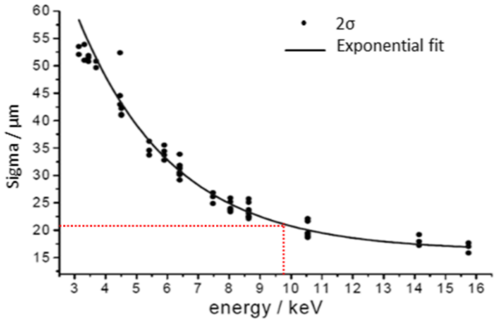

Figure S12: Calibration curve of depth resolution (volume size) fitted with an exponential function.

Table S10: Results of penetration depth and lateral deposition of NGG-1b at variable number of adhesive applications applied over gypsum matrix then gilded over.

Adhesive	Number of applications	Surface Tension* (mN/m)	Gelling Temperature¥ (°C)	Penetration Depth# (µm)	In-depth Distribution (µm)$	Au Foil Delamination
				3D-µXRF	Optical Microscopy	3D-µXRF
Gelatin	1 (3 %)	64.4 ± 0.5	27.9 ± 0.1	19.69 ± 0.24	At surface and through full stratigraphy	Not observed
	4 (12 %)	62.3 ± 0.5	29.8 ± 0.1	20.10 ± 0.23	Deposition in middle part of the cross section	Not observed
	7 (21 %)	60.3 ± 0.5	30.6 ± 0.1	17.99 ± 0.32	At gold-gypsum interface, in middle part of the cross section	Observed with adhesive attached to its lower part. Delamination maxima of ca. 30 µm after 24 h.
	10 (30 %)	N.A.	N.A	17.94 ± 0.24	At gold-gypsum interface, in middle part of the cross section	Observed with adhesive attached to its lower part. Delamination maxima of ca. 70 µm after 24 h.
Water	-	3 ± 1	-	-	-	-

* as reported by Loeb[32] at pH 4.7 and 40 °C N.A.: Not Available
¥ as reported by Hallewell and Peard[34] at pH 4.7 and 25 °C
3D-µXRF depth profiling is used to trace gold penetration depth as a marker element of applied NGG
$ In depth distribution is assessed by OM relying on NGG-1b color light purple color

S5.3 Colorimetric parameters

Table S11. Colorimetric parameters for NGG-1b and NGG-1c applied over the four interfaces

Sample ID			Optical Parameters (± SD)						
			*L**	*a**	*b**	*ΔL**	*Δa**	*Δb**	*ΔE**
Interface with	Raw NGG	NGG-1b	71.93(0.10)	10.62(0.04)	1.27(0.06)	-19.56(0.10)	11.74(0.04)	0.07(0.06)	22.81(0.36)
	Au leaf	b1	90.56 (0.14)	14.48 (1.50)	34.90 (1.12)	1.28 (0.14)	13.60 (1.50)	28.77 (1.12)	31.88 (0.40)
	Fe_2O_3	b2	36.38 (1.97)	24.41 (2.32)	19.87 (1.47)	-53.19 (1.96)	23.53 (2.32)	13.66 (1.47)	59.78 (2.42)
	Fe-rich clay	b3	61.21 (1.77)	19.89 (0.38)	31.17 (0.03)	-28.07 (1.77)	19.01 (0.38)	25.03 (0.03)	42.15 (1.33)
	Gypsum	b4	92.28 (0.05)	0.57 (0.21)	4.71 (0.08)	-2.78 (0.05)	0.59 (0.21)	1.60 (0.08)	3.26 (0.04)
Interface with	Raw NGG	NGG-1c	83.07(0.54)	5.92(0.44)	1.09(0.08)	-8.41(0.54)	7.05(0.44)	-0.10(0.08)	10.97(0.16)
	Au leaf	c1	89.51 (0.15)	7.31 (0.99)	36.19 (3.39)	0.23 (0.15)	6.42 (0.99)	30.05 (3.40)	30.73 (3.52)
	Fe_2O_3	c2	34.90 (2.61)	31.31 (6.89)	25.41 (5.91)	-54.38 (2.61)	30.43 (6.89)	19.28 (5.90)	65.59 (4.45)
	Fe-rich clay	c3	63.76 (0.41)	19.16 (0.14)	31.95 (0.19)	-25.53 (0.41)	18.27 (0.14)	25.82 (0.19)	40.64 (0.24)
	Gypsum	c4	87.80 (2.87)	1.71 (0.89)	2.62 (1.12)	-7.26 (2.87)	1.74 (0.88)	-0.50 (1.12)	7.54 (2.99)

References

1. Zhao, P., Li, N. & Astruc, D. State of the art in gold nanoparticle synthesis. *Coord. Chem. Rev.* **257**, 638–665 (2013).
2. Aryal, S., Remant Bahadur, K. C., Bhattarai, S. R., Prabu, P. & Kim, H. Y. Immobilization of collagen on gold nanoparticles: Preparation, characterization, and hydroxyapatite growth. *J. Mater. Chem.* **16**, 4642–4648 (2006).
3. Dhar, S., Maheswara Reddy, E., Shiras, A., Pokharkar, V. & Prasad, B. L. V. Natural gum reduced/stabilized gold nanoparticles for drug delivery formulations. *Chem. - A Eur. J.* **14**, 10244–10250 (2008).
4. Neupane, M. P. *et al.* Synthesis of gelatin-capped gold nanoparticles with variable gelatin concentration. *J. Nanoparticle Res.* **13**, 491–498 (2011).
5. Jahanshahi, M., Sanati, M. H., Hajizadeh, S. & Babaei, Z. Gelatin nanoparticle fabrication and optimization of the particle size. *Phys. Status Solidi Appl. Mater. Sci.* **205**, 2898–2902 (2008).
6. Lim, S., Gunasekaran, S. & Imm, J. Y. Gelatin-Templated Gold Nanoparticles as Novel Time-Temperature Indicator. *J. Food Sci.* **77**, (2012).
7. Khodashenas, B., Ardjmand, M., Baei, M. S., Rad, A. S. & Khiyavi, A. A. Gelatin–Gold Nanoparticles as an Ideal Candidate for Curcumin Drug Delivery: Experimental and DFT Studies. *J. Inorg. Organomet. Polym. Mater.* **29**, 2186–2196 (2019).
8. Helmholtz-, Z. B. für M. und E. The mySpot beamline at BESSY II. *J. Large-scale Res. Facil.* **2**, 1–4 (2016).
9. Banerjee, S. S. & Chen, D. H. Fast removal of copper ions by gum arabic modified magnetic nano-adsorbent. *J. Hazard. Mater.* **147**, 792–799 (2007).
10. Cretu, C. & Van Der Lingen, E. Coloured gold alloys. *Gold Bull.* **32**, 115–126 (1999).
